# Wide-Dynamic-Range Control of Quantum-Electrodynamic Electron Transfer Reactions in the Weak Coupling Regime

**DOI:** 10.1021/acs.jpclett.4c01265

**Published:** 2024-07-12

**Authors:** Yu-Chen Wei, Liang-Yan Hsu

**Affiliations:** † Institute of Atomic and Molecular Sciences, Academia Sinica, Taipei 106, Taiwan; ‡ Department of Chemistry, National Taiwan University, Taipei 106, Taiwan; ¶ Department of Applied Physics and Science Education, Eindhoven University of Technology, 5600MB Eindhoven, The Netherlands; § National Center for Theoretical Sciences, Taipei 106, Taiwan

## Abstract

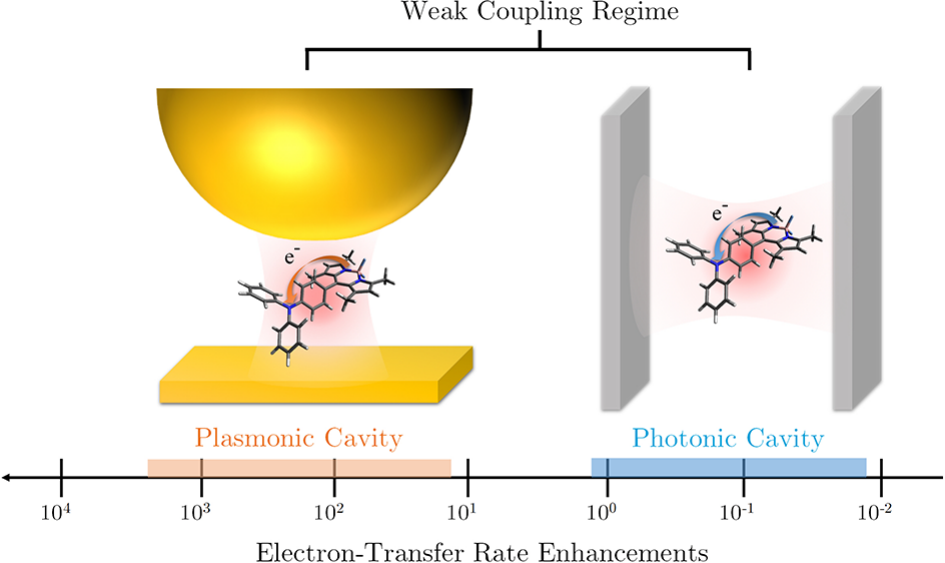

Catalyzing reactions effectively by vacuum fluctuations of electromagnetic fields is a significant challenge within the realm of chemistry. As opposed to most studies based on vibrational strong coupling, we introduce an innovative catalytic mechanism driven by weakly coupled polaritonic fields. Through the amalgamation of macroscopic quantum electrodynamics (QED) principles with Marcus electron transfer (ET) theory, we predict that ET reaction rates can be precisely modulated across a wide dynamic range by controlling the size and structure of nanocavities. Compared to QED-driven radiative ET rates in free space, plasmonic cavities induce substantial rate enhancements spanning the range from 10^3^- to 10-fold. By contrast, Fabry–Perot cavities engender rate suppression spanning the range from 10^–2^- to 10^–1^-fold. This work overcomes the necessity of using strong light–matter interactions in QED chemistry, opening up a new era of manipulating QED-based chemical reactions in a wide dynamic range.

Quantum-electrodynamics (QED) chemistry has become an active field in the past few years due to its potential applications in chemical reactions and optoelectronics.^1−5^ Recent studies have shown that the formation of hybrid light–matter states in the strong coupling regime can modify chemical reactions.^1,2^ Experimentally, the hybrid light–matter states can be utilized to modify the molecular self-assembly,^6−8^ alter the reaction rates^9,10^ and selectively control the reaction pathways.^11,12^ In addition, several theoretical and experimental studies have proposed that it is possible to trigger collective chemical reactions by strongly coupling the molecular ensemble to a confined photon mode.^13−17^ Among the various demonstrations, chemical reactions triggered by formations of hybrid light–matter states require strong light–matter coupling between electromagnetic (EM) modes and molecular transitions. However, maintaining the condition of strong coupling proves challenging due to the constant shifts in the environmental dielectric function during chemical reactions.^1,2,18^ In addition, the reported dynamic ranges of tuning reaction rates via strong vibrational coupling have been found to be limited to less than 1 order of magnitude.^1,3,10^ These issues underscore the limitations in controlling chemical reactions via strong light–matter coupling.

Different from the previous approaches, Semenov et al. proposed that a single cavity mode remarkably enhances electron transfer (ET) rates in the inverted Marcus regime.^19^ In addition, Wei et al. demonstrated that ET reaction rates can be modulated by infinite photonic modes, even in the absence of strong light–matter coupling.^20^ These insightful studies inspire us to ask a question: Is it possible to effectively control chemical reaction rates over several orders of magnitude without relying on strong light–matter coupling? To answer this question, we present a feasible weak-coupling approach to manipulate ET rates via tuning cavity sizes. To quantitatively describe QED effects on ET reactions in a medium, we develop a macroscopic QED version of ET (mQED-ET) theory by considering the influence of infinite polaritonic modes. According to this approach, we study how vacuum fluctuations of infinite polaritonic modes influence the radiative charge recombination process in a donor–acceptor dyad. This study offers new insights into the underlying mechanisms and experimental design principles for QED-controlled chemical reactions in the weak light–matter coupling regime.

To properly describe the light–matter interaction in a dispersive and absorbing dielectric environment, we start from the length-gauge Hamiltonian in the framework of macroscopic QED.^21−23^
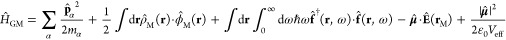
1*m*_α_ and **p̂**_α_ indicate the mass and the momentum operator of the α^th^ particle, respectively. ρ̂_M_(**r**) represents a charge density operator for the molecule. ϕ̂_M_(**r**) corresponds to molecular Coulomb potential operator. **f̂**^†^(**r**,ω) (**f̂**(**r**,ω)) is the creation (annihilation) operator for bosonic vector fields (polariton) in macroscopic QED.^21−23^ The term **μ̂**·**Ê**(**r**_M_) describes the light–matter interaction in the electric-dipole form, where **μ̂** and **Ê**(**r**_M_) represent the dipole moment operator and the electric field operator, respectively. The last term is the dipole self-energy, where *V*_eff_ is the effective mode volume of the transverse EM fields. The detailed discussion about eq 1 and its derivation from the minimal coupling Hamiltonian are elaborated in our previous study.^24^ Note that the length gauge Hamiltonian retains intramolecular Coulomb interactions and electromagnetic vacuum fluctuations caused by the transversal polaritonic degrees of freedom in a medium. The former allows us to describe the Coulomb electronic coupling and the vibronic coupling in Marcus theory, and the latter enables us to depict the medium-assisted QED effects on ET processes.

To obtain effective electronic coupling, we develop the model Hamiltonian considering electronic, vibrational and polaritonic degrees of freedom (see section S1 of the Supporting Information). Following the standard molecular ET theory,^19,25^ we consider a two-state molecular model in the electronic subspace with a harmonic vibrational bath. To consider the effects of permanent dipoles and dipole self-energy on light–matter coupling, we adopt the unitary transformation along polaritonic coordinates via *Û*_pol_ (details in section S2).
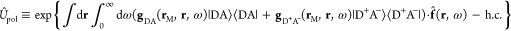
2DA (D^+^A^–^) indicates the electronic initial (final) state. **g**_DA_(**r**_M_,**r**,ω) and **g**_D^+^A^–^_(**r**_M_,**r**,ω) are the unit polaritonic displacements induced by the permanent dipoles of DA and D^+^A^–^ states, respectively. Next, we perform the small-polaron transformation , where *m*_vib_ represents the number of harmonic vibrational modes. The *u*^th^ vibrational mode possesses the frequency ω_vib,u_ with the vibrational creation (annihilation) operator *b̂*_*u*_^†^ (*b̂*_*u*_) and the vibrational Huang–Rhys factor *S*_vib,u_ (details in section S3). After the series of unitary transformations, we obtain the working Hamiltonian .
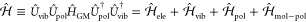
3
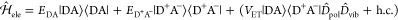
4
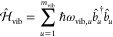
5
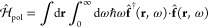
6
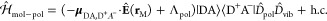
7, ,  and  correspond to the transformed electronic Hamiltonian, the transformed vibrational Hamiltonian, the transformed polaritonic Hamiltonian and the transformed light–molecule interaction, respectively. *E*_DA_ (*E*_D^+^A^–^_) indicates the electronic energy of the corresponding states. *V*_ET_ is the Coulomb electronic coupling. **μ**_DA,*D*^+^*A*^–^_ indicates the transition dipole moment between DA state and D^+^A^–^ state. Λ_pol_ is the reorganization dipole self-coupling induced by permanent dipole moment change and dipole self-energy.  indicates the vibrational displacement operator. *D̂*_pol_ corresponds to the polaritonic displacement operator expressed as *D̂*_pol_ ≡ exp{−∫ d**r**∫_0_^∞^ dωΔ**g**(**r**_M_,**r**,ω)·**f̂**(**r**,ω) + h.c.}, where Δ**g**(**r**_M_,**r**,ω) = **g**_D^+^A^–^_(**r**_M_,**r**,ω) – **g**_DA_(**r**_M_,**r**,ω) represents the unit polaritonic displacements caused by the permanent dipole change.^24^ According to eqs 3 and 6, we can define the effective electronic coupling  by collecting all the electronic off-diagonal terms together.
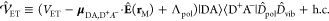
8Given the coupling in eq 8, one can use the Fermi’s golden rule to evaluate ET rate in the electronic weak-coupling approximation and the light–matter weak-coupling approximation. Note that the breakdown of the weak-coupling approximation will lead to the Rabi oscillation in the population dynamics.^26^ Next, based on the same approximations in QED-ET theory including the Condon approximation, the short-time approximation, and the low-vibrational-frequency limit,^20^ we derive an explicit expression for the total ET rate *k*_ET_ as
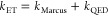
9The details of how to derive the mQED-ET theory from Fermis golden rule can be found in section S4. *k*_Marcus_ corresponds to the Marcus nonradiative ET rate,^27^ which is defined in eq S59. *k*_QED_ is the QED-driven radiative ET rate. eq 9 conveys three significant insights. First, the mQED-ET theory integrates both the Marcus ET theory and the QED-driven ET mechanism into a unified theoretical framework. Second, in the weak light–matter coupling regime, the QED-driven ET mechanism can effectively catalyze ET reactions, particularly when the Marcus nonradiative ET process is negligible (*k*_Marcus_ ≪ *k*_QED_). Third, the ET reactions can be categorized into two types: Nonradiative ET driven by electrostatic Coulomb interaction (*k*_Marcus_) and radiative ET driven by vacuum fluctuations (*k*_QED_). The former has been extensively studied in previous studies,^27,28^ while the latter has not been systematically explored, which is the primary objective of this study.

Next, *k*_QED_ is expressed as the integral of polaritonic spectral density *J*_pol_(ω) and vibronic density of states (DOS) ρ_vib_(ω) in the following form:
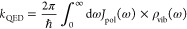
10where *J*_pol_(ω) represents the polaritonic spectral density. Since the contributions from Λ_pol_ and *D̂*_pol_ on light–matter coupling strength are negligible,^24^*J*_pol_ (ω) can be reduced to
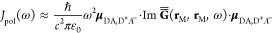
11 is the imaginary part of the local dyadic Green’s function, which is associated with the local photonic DOS (local density of optical states).^29,30^ Note that the integral of *J*_pol_(ω) corresponds to the square of the light-matter coupling strength and ρ_vib_(ω) describes vibronic transitions during an ET process.^20^ According to eq 10, we propose a generalized version of “electron-transfer overlap” corresponding to the spectral overlap of the polaritonic spectral density *J*_pol_(ω) (the red curve) and the vibronic DOS ρ_vib_(ω) (the blue curve), whose physical pictures are shown in Figure 1. If we consider the condition of a single photonic mode, the photonic DOS behaves as a single-peak delta function (Figure 1a) and eq 9 can be reduced to the cavity QED version of ET rates, as derived by Semenov and Nitzan under the condition of slow electron and fast cavity mode^19^ (see section S5). Next, in the case of a dielectric environment corresponding to free space, the infinite photonic modes exhibit a cubic frequency dependence in the photonic DOS (Figure 1b), aligning with the vacuum QED-ET theory (see section S6).^20^ In this work, *J*_pol_(ω) can describe the polaritonic DOS in an arbitrary dielectric environment (Figure 1c), encompassing the cavity QED-ET theory and the vacuum QED-ET theory as its special cases.

**Figure 1:**
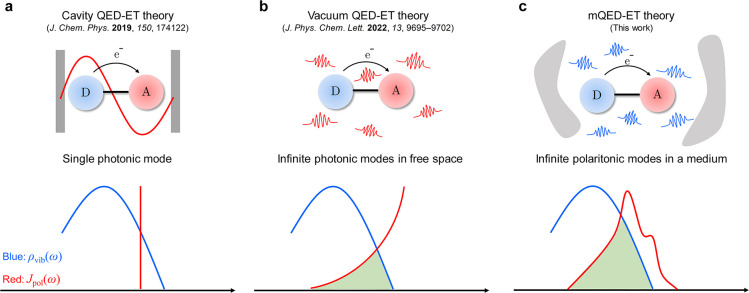
Schematic illustration of spectral overlaps for the radiative ET process. (a) Cavity QED-ET theory developed by Semenov and Nitzan.^19^ (b) Vacuum QED-ET theory developed by Wei and Hsu.^20^ (c) mQED-ET theory developed in this work. The blue curve represents the vibronic DOS ρ_vib_(ω). The red curve represents the photonic/polaritonic spectral density *J*_pol_(ω). The green diagonal stripes represent the concept of electron-transfer overlap, i.e., the spectral overlap between the vibronic DOS and the polaritonic spectral density. The *x*-axis indicates the photonic frequency.

The niches of eq 10 are elaborated as follows. First, *J*_pol_(ω) can be evaluated in an arbitrary dielectric environment without free parameters based on analytical methods (e.g., the Fresnel method and the Mie theory) or computational electrodynamics packages.^26,31,32^ This feasibility can help experimentalists to predict and analyze *k*_QED_. Second, eq 10 suggests that the manipulation of ET rates in the weak light–matter coupling regime is possible by adjusting electron-transfer overlap, which can be achieved by modifying cavity structures during experiments. In simpler terms, the mQED-ET theory can be used by experimentalists to design photonic/plasmonic structures for controlling QED-driven chemical reactions. Note that the parts of ET reactions in the normal Marcus region are endothermic, leading to the emergence of pathways starting from high levels of initial polaritonic states. In this case, our theory cannot accurately describe QED-ET rates because we made the low-temperature approximation for the initial polaritonic states to exclude these pathways. Nevertheless, the main findings in this study do not change because such an approximation is reasonable for ET reactions in the highly inverted region (See our ET system afterward).

In order to manipulate molecular ET rates via QED effects, it is essential for the QED-driven ET rates *k*_QED_ to be significantly higher than the Marcus nonradiative ET rates *k*_Marcus_. According to the QED-ET theory, such a situation usually arises in the highly inverted regime.^20^ Guided by this principle, our investigation focuses on examining the impact of QED effects on the process of radiative charge recombination in a donor–acceptor dyad BODIPY-TPA based on the fluorescent BODIPY functionalized with the triphenylamine (TPA) (Figure 2a). In terms of charge recombination, the electron density undergoes migration from the BODIPY moiety to the TPA moiety, as revealed by the natural transition orbital analysis (Figure 2b). It is noteworthy that the remarkable photoluminescent quantum yield (PLQY = 12.5%) and radiative rate (*k* = 2 × 10^7^ s^–1^) supports the occurrence of a substantial radiative ET process in the BODIPY-TPA system.^33^ This aspect makes it particularly well-suited for manipulation through light–matter interactions.

**Figure 2:**
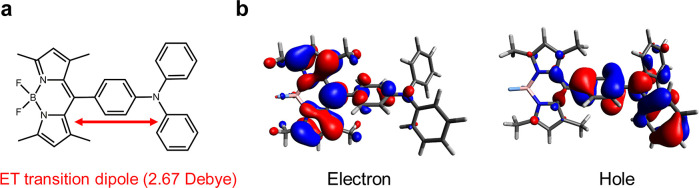
Candidate molecule for controlling the ET reaction via weak light–matter interaction. (a) Chemical structure of BODIPY-TPA. (b) Natural transition orbital analysis of the donor–acceptor dyad BODIPY-TPA. The red double arrow in panel a represents the direction of the ET transition dipole of the charge-transfer transition. The molecular geometry in panel b is optimized in the charge-transfer state. See Computational Methods for detailed information on the quantum chemical calculation.

To regulate charge recombination rates under the weak light–matter coupling regime, two types of cavities are designed. The first type is a plasmonic cavity comprising a dielectric-coated gold surface and a gold nanosphere (Figure 3a). The second type is a photonic cavity, specifically a Fabry–Perot cavity, constructed by sandwiching a dielectric layer between two thin films of silver (Figure 3b). Note that the two kinds of cavities mentioned above are widely used in the fields of nanophotonics, plasmonics and polariton chemistry for developing significant light–matter interaction.^3,34,35^ In addition, molecules in these cavities are under a weak light–matter coupling regime owing to two reasons. First, the molecular transition energy greatly deviates from the peak energy of the *J*_pol_(ω) (Figure S1), leading to the poor ET spectral overlap. Second, the photonic dissipation estimated from the peak width of *J*_pol_(ω) and the electronic energy gap (2.0 eV) majorly exceeds the light–matter coupling strength (Figure S1). The detailed methods of evaluating light–matter coupling strength and photonic dissipation are shown in our previous studies.^24,36,37^ Worth to mention, the energy gap dependence of the ET rates in the two nanocavities reveals that *k*_QED_ significantly surpasses *k*_Marcus_ in the charge recombination process of BODIPY-TPA (Figure S2), supporting the rationale for disregarding *k*_Marcus_. To modify QED-driven ET rates, adjustments are made to the gap distance (H) between the metal surface and the nanosphere in the plasmonic cavity, as well as the length (L) of the cavity in the photonic cavity. These parameters can be mechanically tuned in experiments by changing the positions of mirrors or a tip electrode.^1,38^ It is worth noting that the long-wavelength approximation remains applicable since the cavity sizes here are significantly larger than the molecular size.^39^ Based on the mQED-ET theory, the rate enhancements *k*_QED,m_/*k*_QED, 0_ are evaluated, where *k*_QED,m_ and *k*_QED, 0_ represent the radiative ET rates in a medium and vacuum, respectively (Figure 3c and 3d). The results demonstrate that the plasmonic cavity causes ET rate enhancements ranging from 10^3^–10^1^, whereas the photonic cavity suppresses the ET rates by a factor of 10^–2^–10^–1^. The dynamic ranges of modulating radiative ET rates in both nanocavities reach an impressive 2 orders of magnitude, which far exceeds the rate change induced by vibrational strong coupling.^1,3,10^ Furthermore, the plasmonic cavity acts as an accelerator, while the photonic cavity serves as a decelerator for QED-driven ET reactions. This interplay enables us to achieve a further wide dynamic range of the mQED-ET theory. Such wide tunability of QED-driven ET rates holds significant potential for their practical application in QED-based chemical reactions. In addition, we evaluate the ET rate enhancements in a single gold nanosphere system, which is common in various plasmonic applications,^40,41^ to compare their rate enhancement with the ones in the plasmonic cavity in Figure 3a and 3c. The rate enhancements range from 10^2^ to 10 for molecular heights within 4 to 10 nm (Figure S3), which are less significant than those in the plasmonic cavity due to reduced field confinements.

**Figure 3:**
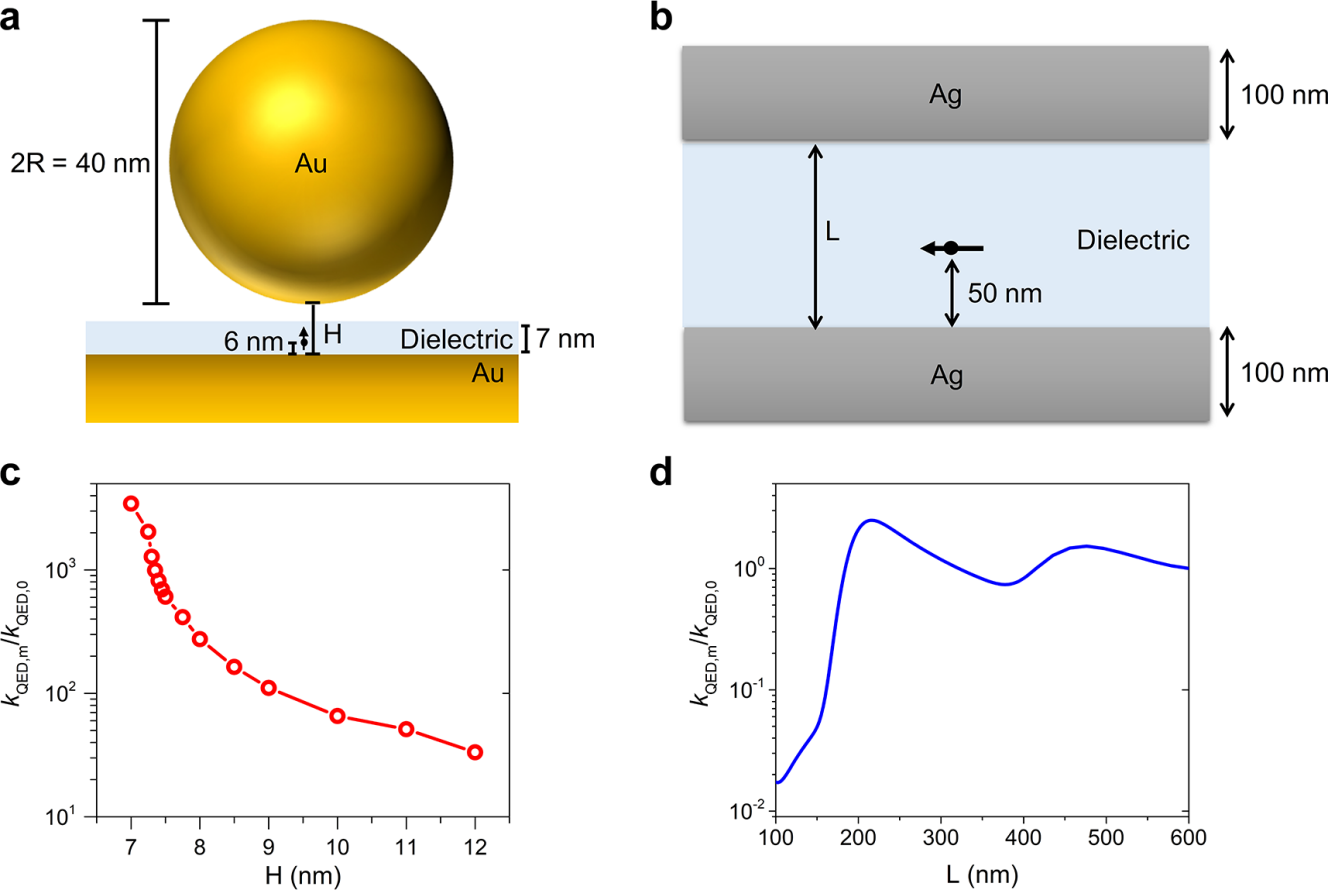
Manipulating QED-driven ET rates via modifying cavity structures. (a) Schematic illustration of the plasmonic cavity. (b) Schematic illustration of the photonic cavity. The black arrow indicates the molecular transition dipole’s position (directly under the sphere) and direction. The molecule is located 6 nm from the metal surface, and the direction of the transition dipole is perpendicular to the metal surface. H represents the gap between the metal surface and the nanosphere. L indicates the cavity length. The dielectric layers in panels a and b are nonabsorbing with a constant refractive index *n* = 1.5. (c) QED-driven ET rate enhancements in the plasmonic cavity. (d) QED-driven ET rate enhancements in the photonic cavity. *k*_QED,m_ and *k*_QED,0_ indicate the radiative ET rates in a medium and free space, respectively. Note that the hollow circles in panel c represent the data points calculated by the computational electrodynamics packages, while the blue lines in *k*_QED,0_ are evaluated by the analytical method.^20^ See Computational Methods for the detailed information on evaluating *k*_QED_.

To better reflect practical experimental conditions, we investigate the ET rate enhancements for a randomly oriented molecule in the plasmonic/photonic cavity, as illustrated in Figure S4. The findings reveal that, even with random molecular orientations, the notable ET rate enhancements persist in the plasmonic cavity. In contrast, the pronounced ET rate suppression disappears in the photonic cavity when a molecule is randomly oriented due to the involvement of the TE modes. These results indicate the necessity for precise control of experimental conditions. In addition, if the cavity length L becomes several nanometers, the ET rate enhancements in the photonic cavity will be similar to the results in the plasmonic cavity due to the dominant effects from surface plasmon polariton. Moreover, to characterize the effective region where the reaction rate can be significantly modified, we evaluate the ET rate enhancements at different horizontal positions of a single vertical dipole positioned 6 nm above the surface (Figure S5). Given the radial symmetry of the plasmonic cavity, we focus on the ET rate enhancements along the horizontal radial direction. The results show that ET rate enhancements remain 3 orders of magnitude higher at a horizontal displacement of 4.25 nm from the position directly beneath the sphere, indicating a significant impact on a specific region of molecules. Notably, the effects of light–matter coupling induced by permanent dipole changes and dipole self-energy can be neglected in the calculation of *k*_QED_ since these effects contribute corrections of less than 1.3% on QED-driven ET rate enhancements (Figure S6). Moreover, since the nanocavities affect both the spectral density and the light out-coupling, the weak light–matter coupling would influence the emission spectra of the QED-ET processes.

The above phenomena can be explained through the analysis of the electron-transfer overlap (Figure 4). In the plasmonic cavity, the significant increase in QED-driven ET rates is attributed to the large polaritonic spectral density *J*_pol_(ω) contributed by surface plasmon polaritons. Moreover, replacing gold with silver as the material for the plasmonic cavity results in a slight reduction in the ET rate enhancements. This reduction is attributed to the smaller electron-transfer overlap between vibronic DOS and polaritonic spectral density from the silver plasmonic cavity (Figure S7). On the other hand, the orthogonality between the dipole and the transverse electric (TE) modes in the photonic cavity causes a reduction in the *J*_pol_(ω) and consequently a decrease in the corresponding QED-driven ET rates.^29^ Note that the QED-driven ET rate suppression here should not be confused with the events occurring within the strong light–matter coupling regime.^3,11,42^ The criteria for achieving strong coupling require a high photonic DOS. In contrast, ET rate suppression here results from the lower photonic DOS within the photonic cavity as compared to the ones in a vacuum. In addition, the results align with the Purcell effects, as both radiative ET rates and spontaneous emission rates are influenced by the local polaritonic/photonic DOS.^43,44^ Despite the similarities between the Purcell effect and QED-driven ET, they are fundamentally different. First, QED-driven ET involves significant displacements of electron/nucleus interacting with vacuum photonic/polaritonic fluctuations, whereas the Purcell effect, which pertains to spontaneous emission, primarily focuses on the interaction between local electronic transitions and the photonic DOS. Second, in QED-driven ET, the initial and final states correspond to charge separation and charge recombination, respectively. The two electronic states are not orthogonal, and they are diabatic states, i.e., the two states have electronic couplings. On the other hand, in the Purcell effect, the initial and final states correspond to excited and ground states, respectively. The two electronic states are orthogonal, i.e., no electronic couplings between the two states. Furthermore, the polariton-assisted ET rates also exhibit a near absence of activation energy (Figure S8), attributed to a mechanism analogous to that explained in the QED-ET theory.^20^ Moreover, the QED-driven ET processes might be negligible in several ET systems due to small ET transition dipole moments (less than 0.1 D)^45^ or significant nonradiative processes.^28^ According to eq 11, the conditions for substantial QED-driven ET processes comprise a nonzero|**μ**_DA,*D*^+^*A*^–^_|, an exothermic energy landscape (in an inverted regime) and a high polaritonic DOS (). Such ET reactions are common in photoinduced ET processes, with significant implications for applications like solar cells,^46,47^ organic light-emitting diodes,^48^ and photocatalysis.^49,50^

**Figure 4:**
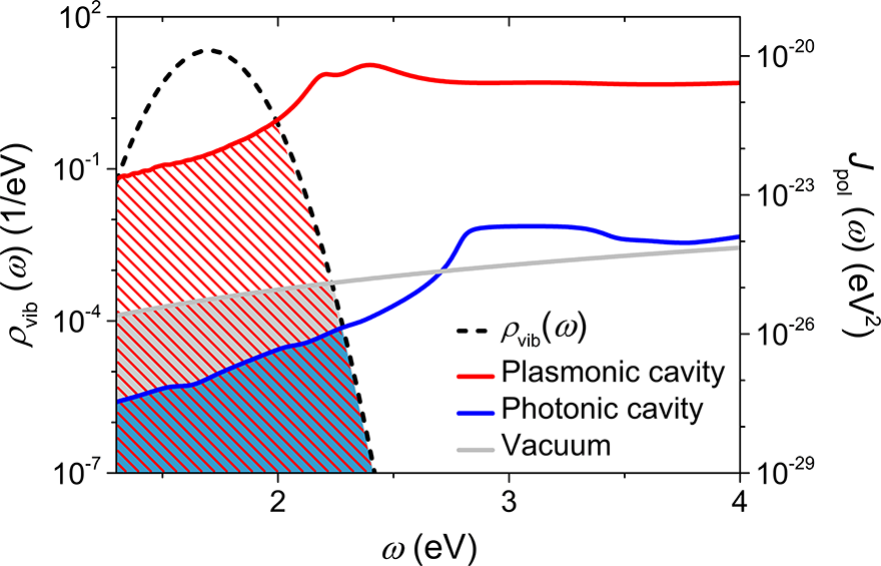
Electron-transfer overlap in the plasmonic and photonic cavities. The stripes and the shaded regions represent the electron-transfer overlaps. The following parameters for the two cavities are applied: H = 7 nm and L = 100 nm. The parameters of ρ_vib_(ω) follow those in Figure 3.

Our key findings are summarized as follows. First, the manipulation of chemical reaction rates through weak light–matter interaction has been demonstrated, which is different from the previous methods that require strong light–matter coupling conditions. Second, we have derived a mQED-version ET theory and generalized the concept of electron-transfer overlap: the spectral overlap of the polaritonic spectral density *J*_pol_(ω) and vibronic DOS ρ_vib_(ω), which elaborates the interplay of polaritonic modes and ET reactions. This breakthrough allows us to quantify QED-driven ET rates in an arbitrary absorbing and dispersive medium. Third, our theory predicted that different types of cavities cause opposite effects on molecular charge recombination rates. The underlying mechanism can be understood through the physical picture of electron-transfer overlap. Fourth, the QED-driven ET rates show great adjustability via tuning sizes of nanocavities, giving rise to the remarkable dynamic ranges spanning 2 orders of magnitude within the designed cavities. Note that the wide dynamic range shown in weak-coupling QED-ET reactions surpasses the range observed in chemical reactions controlled by vibrational strong coupling.^1,3,10^ This exceptional capability to manipulate ET rates possesses substantial potential for controlling chemical reactions through QED effects. Worth to mention, the mQED-ET theory considers the single-molecule regime, while most experiments deal with a molecular ensemble. Therefore, the effects of molecular ensembles on QED-driven ET rates deserve further investigation. In addition, the influence of the weak light–matter coupling on other photophysical processes is expected to be minimal owing to the negligible QED-driven rates of internal conversion and intersystem crossing.^51^ In summary, this study offers a generalized approach to predict QED-driven chemical reactions under weak light–matter interactions. We anticipate that this work will inspire and support novel developments of QED-based chemical reactions.

## Computational Methods

*Quantum Chemical Calculation*. We performed density functional theory (DFT) calculations at the ωB97XD/6-31+g(d) level with the Gaussian 16 program.^52^ The geometries of the ground state and the excited states were optimized using DFT and time-dependent DFT (TD-DFT), respectively. The polarizable continuum method (PCM) model was used to simulate the solvent environment (chloroform).^53^ The natural transition orbitals were calculated by the Multiwfn program.^54^ Similar calculation methods has been reported in the previous study.^55^

*Numerical Parameters for Evaluating k_QED_*. We use eqs 10 and 11 as the working equations to evaluate *k*_QED_. To evaluate the  of the plasmonic cavity, we applied the Lumerical FDTD Solutions^56^ to obtain the Purcell factor *F* and utilize the relation:^29^
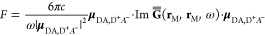
12In addition, we applied the Fresnel method and the Mie’s theory to analytically evaluate the  of the photonic cavity and the gold nanosphere, respectively.^23,26,31,36^ To evaluate the integral in eq 10, it is necessary to introduce a cutoff frequency into the upper bounds of the integral. According to our previous work,^20^ the cutoff frequency should be related to the size of a molecule to fulfill the long-wavelength approximation. In this study, the upper bound and the lower bound frequencies are set as 6.2 and 1.23 eV for all systems (including free space). Higher upper bound frequency has been tested (17 eV), which gives rise to the rate enhancement deviation less than 2 fold. In addition, different lower bound frequencies do not significantly change the numerical results due to low local polaritonic DOS in the low-frequency regime. Considering the effects of permanent dipole change and dipole-self-energy, we apply eq S67 and the following parameters: permanent dipole change |*Δ**μ***|= 3.50 D according to our quantum chemical calculation and Coulomb electronic coupling *V*_ET_ = 89.3 meV.^33^ The directions of permanent dipole change and transition dipole are the same.^33^

The dielectric functions of gold from Olson, Lynch and Weaver^57^ and silver from Johnson and Christy^58^ were used for the plasmonic and the photonic cavity, respectively. On the other hand, the parameters of ρ_vib_(ω) under the limit of low vibrational frequency include the energy gap Δ*E*, temperature *T* and reorganization energy *E*_r_, which can be obtained from the reported experimental results.^33^ Δ*E* = 1.96 eV corresponds to the peak energy of the steady-state emission spectra. *T* = 300 *K* represents room temperature. *E*_r_ = 0.26 eV can be estimated from the Stokes shift of the steady-state spectra.^59,60^

## Supporting Information Available

Model Hamiltonian, unitary transformation along polaritonic coordinates, small-polaron transformation, derivation of eqs 9 and 10, conversion of mQED-ET theory into cavity QED-ET theory, conversion of mQED-ET theory into vacuum QED-ET theory, supporting figures, and supporting references (PDF)

## References

[ref1] Nagarajan K.; Thomas A.; Ebbesen T. W. Chemistry under Vibrational Strong Coupling. J. Am. Chem. Soc. 2021, 143, 16877–16889. 10.1021/jacs.1c07420. 34609858

[ref2] Ebbesen T. W. Hybrid Light-Matter States in a Molecular and Material Science Perspective. Acc. Chem. Res. 2016, 49, 2403–2412. 10.1021/acs.accounts.6b00295. 27779846

[ref3] Garcia-Vidal F. J.; Ciuti C.; Ebbesen T. W. Manipulating Matter by Strong Coupling to Vacuum Fields. Science 2021, 373, 0336 10.1126/science.abd0336. 34244383

[ref4] Li T. E.; Cui B.; Subotnik J. E.; Nitzan A. Molecular Polaritonics: Chemical Dynamics under Strong Light-Matter Coupling. Annu. Rev. Phys. Chem. 2022, 73, 43–71. 10.1146/annurev-physchem-090519-042621. 34871038

[ref5] George J.; Singh J. Polaritonic Chemistry: Band-Selective Control of Chemical Reactions by Vibrational Strong Coupling. ACS Catal. 2023, 13, 2631–2636. 10.1021/acscatal.2c05201.

[ref6] Joseph K.; Kushida S.; Smarsly E.; Ihiawakrim D.; Thomas A.; Paravicini-Bagliani G. L.; Nagarajan K.; Vergauwe R.; Devaux E.; Ersen O.; et al. Supramolecular Assembly of Conjugated Polymers under Vibrational Strong Coupling. Angew. Chem., Int. Ed. 2021, 60, 19665–19670. 10.1002/anie.202105840. 34255910

[ref7] Hirai K.; Ishikawa H.; Chervy T.; Hutchison J. A.; Uji-i H. Selective Crystallization via Vibrational Strong Coupling. Chem. Sci. 2021, 12, 11986–11994. 10.1039/D1SC03706D. 34667564 PMC8457383

[ref8] Sandeep K.; Joseph K.; Gautier J.; Nagarajan K.; Sujith M.; Thomas K. G.; Ebbesen T. W. Manipulating the Self-Assembly of Phenyleneethynylenes under Vibrational Strong Coupling. J. Phys. Chem. Lett. 2022, 13, 1209–1214. 10.1021/acs.jpclett.1c03893. 35089035

[ref9] Vergauwe R. M.; Thomas A.; Nagarajan K.; Shalabney A.; George J.; Chervy T.; Seidel M.; Devaux E.; Torbeev V.; Ebbesen T. W. Modification of Enzyme Activity by Vibrational Strong Coupling of Water. Angew. Chem., Int. Ed. 2019, 58, 15324–15328. 10.1002/anie.201908876. PMC6856831 31449707

[ref10] Ahn W.; Triana J.; Recabal F.; Herrera F.; Simpkins B. Modification of Ground State Chemical Reactivity via Light-Matter Coherence in Infrared Cavities. Science 2023, 380, 1165–1168. 10.1126/science.ade7147. 37319215

[ref11] Thomas A.; Lethuillier-Karl L.; Nagarajan K.; Vergauwe R. M.; George J.; Chervy T.; Shalabney A.; Devaux E.; Genet C.; Moran J.; et al. Tilting a Ground-State Reactivity Landscape by Vibrational Strong Coupling. Science 2019, 363, 615–619. 10.1126/science.aau7742. 30733414

[ref12] Sau A.; Nagarajan K.; Patrahau B.; Lethuillier-Karl L.; Vergauwe R. M.; Thomas A.; Moran J.; Genet C.; Ebbesen T. W. Modifying Woodward-Hoffmann Stereoselectivity under Vibrational Strong Coupling. Angew. Chem., Int. Ed. 2021, 60, 5712–5717. 10.1002/anie.202013465. PMC7986062 33305864

[ref13] Herrera F.; Spano F. C. Cavity-Controlled Chemistry in Molecular Ensembles. Phys. Rev. Lett. 2016, 116, 238301 10.1103/PhysRevLett.116.238301. 27341263

[ref14] Sidler D.; Schäfer C.; Ruggenthaler M.; Rubio A. Polaritonic Chemistry: Collective Strong Coupling Implies Strong Local Modification of Chemical Properties. J. Phys. Chem. Lett. 2021, 12, 508–516. 10.1021/acs.jpclett.0c03436. 33373238 PMC7928910

[ref15] Li T. E.; Nitzan A.; Subotnik J. E. Collective Vibrational Strong Coupling Effects on Molecular Vibrational Relaxation and Energy Transfer: Numerical Insights via Cavity Molecular Dynamics Simulations. Angew. Chem., Int. Ed. 2021, 60, 15533–15540. 10.1002/anie.202103920. 33957010

[ref16] Gómez J. A.; Vendrell O. Vibrational Energy Redistribution and Polaritonic Fermi Resonances in the Strong Coupling Regime. J. Phys. Chem. A 2023, 127, 1598–1608. 10.1021/acs.jpca.2c08608. 36758162

[ref17] Du M.; Poh Y. R.; Yuen-Zhou J. Vibropolaritonic Reaction Rates in the Collective Strong Coupling Regime: Pollak-Grabert-Hänggi Theory. J. Phys. Chem. C 2023, 127, 5230–5237. 10.1021/acs.jpcc.3c00122.

[ref18] Simpkins B. S.; Dunkelberger A. D.; Owrutsky J. C. Mode-Specific Chemistry through Vibrational Strong Coupling (or A Wish Come True). J. Phys. Chem. C 2021, 125, 19081–19087. 10.1021/acs.jpcc.1c05362.

[ref19] Semenov A.; Nitzan A. Electron Transfer in Confined Electromagnetic Fields. J. Chem. Phys. 2019, 150, 174122 10.1063/1.5095940. 31067889

[ref20] Wei Y.-C.; Hsu L.-Y. Cavity-Free Quantum-Electrodynamic Electron Transfer Reactions. J. Phys. Chem. Lett. 2022, 13, 9695–9702. 10.1021/acs.jpclett.2c02379. 36219782

[ref21] Gruner T.; Welsch D.-G. Green-Function Approach to the Radiation-Field Quantization for Homogeneous and Inhomogeneous Kramers-Kronig Dielectrics. Phys. Rev. A 1996, 53, 1818 10.1103/PhysRevA.53.1818. 9913077

[ref22] Dung H. T.; Knöll L.; Welsch D.-G. Three-Dimensional Quantization of the Electromagnetic Field in Dispersive and Absorbing Inhomogeneous Dielectrics. Phys. Rev. A 1998, 57, 3931 10.1103/PhysRevA.57.3931.

[ref23] Buhmann S. Y. Dispersion Forces I: Macroscopic quantum electrodynamics and ground-state Casimir, Casimir–Polder and Van Der Waals forces; Springer, 2013.

[ref24] Wei Y.-C.; Hsu L.-Y. Polaritonic Huang-Rhys Factor: Basic Concepts and Quantifying Light-Matter Interactions in Media. J. Phys. Chem. Lett. 2023, 14, 2395–2401. 10.1021/acs.jpclett.3c00065. 36856331

[ref25] Nitzan A. Chemical Dynamics in Condensed Phases: Relaxation, Transfer and Reactions in Condensed Molecular Systems; Oxford University Press, 2006.

[ref26] Wang S.; Scholes G. D.; Hsu L.-Y. Coherent-to-Incoherent Transition of Molecular Fluorescence Controlled by Surface Plasmon Polaritons. J. Phys. Chem. Lett. 2020, 11, 5948–5955. 10.1021/acs.jpclett.0c01680. 32619095

[ref27] Marcus R. A. Electron Transfer Reactions in Chemistry. Theory and Experiment. Rev. Mod. Phys. 1993, 65, 599 10.1103/RevModPhys.65.599.

[ref28] Kumpulainen T.; Lang B.; Rosspeintner A.; Vauthey E. Ultrafast Elementary Photochemical Processes of Organic Molecules in Liquid Solution. Chem. Rev. 2017, 117, 10826–10939. 10.1021/acs.chemrev.6b00491. 27957848

[ref29] Novotny L.; Hecht B. Principles of Nano-Optics; Cambridge University Press, 2012.

[ref30] Ter Huurne S. E.; Peeters D. B.; Sánchez-Gil J. A.; Rivas J. G. Direct Measurement of the Local Density of Optical States in the Time Domain. ACS photonics 2023, 10, 2980–2986. 10.1021/acsphotonics.3c00781. 37602289 PMC10436706

[ref31] Lee M.-W.; Hsu L.-Y. Controllable Frequency Dependence of Resonance Energy Transfer Coupled with Localized Surface Plasmon Polaritons. J. Phys. Chem. Lett. 2020, 11, 6796–6804. 10.1021/acs.jpclett.0c01989. 32787214

[ref32] Wei Y.-C.; Lee M.-W.; Chou P.-T.; Scholes G. D.; Schatz G. C.; Hsu L.-Y. Can Nanocavities Significantly Enhance Resonance Energy Transfer in a Single Donor-Acceptor Pair?. J. Phys. Chem. C 2021, 125, 18119–18128. 10.1021/acs.jpcc.1c04623.

[ref33] Buck J. T.; Wilson R. W.; Mani T. Intramolecular Long-Range Charge-Transfer Emission in Donor-Bridge-Acceptor Systems. J. Phys. Chem. Lett. 2019, 10, 3080–3086. 10.1021/acs.jpclett.9b01269. 31117690

[ref34] Vasa P.; Lienau C. Strong Light-Matter Interaction in Quantum Emitter/Metal Hybrid Nanostructures. ACS Photonics 2018, 5, 2–23. 10.1021/acsphotonics.7b00650.

[ref35] Rivera N.; Kaminer I. Light-Matter Interactions with Photonic Quasiparticles. Nat. Rev. Phys. 2020, 2, 538–561. 10.1038/s42254-020-0224-2.

[ref36] Wang S.; Scholes G. D.; Hsu L.-Y. Quantum Dynamics of a Molecular Emitter Strongly Coupled with Surface Plasmon Polaritons: A Macroscopic Quantum Electrodynamics Approach. J. Chem. Phys. 2019, 151, 014105 10.1063/1.5100014. 31272186

[ref37] Wang S.; Chuang Y.-T.; Hsu L.-Y. Simple but Accurate Estimation of Light-Matter Coupling Strength and Optical Loss for a Molecular Emitter Coupled with Photonic Modes. J. Chem. Phys. 2021, 155, 134117 10.1063/5.0060171. 34624977

[ref38] Lorente N.; Rurali R.; Tang H. Single-Molecule Manipulation and Chemistry with the STM. J. Condens. Matter Phys. 2005, 17, S1049 10.1088/0953-8984/17/13/003.

[ref39] Neuman T.; Esteban R.; Casanova D.; García-Vidal F. J.; Aizpurua J. Coupling of Molecular Emitters and Plasmonic Cavities beyond the Point-Dipole Approximation. Nano Lett. 2018, 18, 2358–2364. 10.1021/acs.nanolett.7b05297. 29522686

[ref40] Dreaden E. C.; Alkilany A. M.; Huang X.; Murphy C. J.; El-Sayed M. A. The Golden Age: Gold Nanoparticles for Biomedicine. Chem. Soc. Rev. 2012, 41, 2740–2779. 10.1039/C1CS15237H. 22109657 PMC5876014

[ref41] Li N.; Zhao P.; Astruc D. Anisotropic Gold Nanoparticles: Synthesis, Properties, Applications, and Toxicity. Angew. Chem., Int. Ed. 2014, 53, 1756–1789. 10.1002/anie.201300441. 24421264

[ref42] Thomas A.; George J.; Shalabney A.; Dryzhakov M.; Varma S. J.; Moran J.; Chervy T.; Zhong X.; Devaux E.; Genet C.; et al. Ground-State Chemical Reactivity under Vibrational Coupling to the Vacuum Electromagnetic Field. Angew. Chem., Int. Ed. 2016, 55, 11462–11466. 10.1002/anie.201605504. PMC5113700 27529831

[ref43] Purcell E. M. Confined Electrons and Photons: New Physics and Applications; Springer, 1995; p 839.

[ref44] Vahala K. J. Optical Microcavities. Nature 2003, 424, 839–846. 10.1038/nature01939. 12917698

[ref45] Shin Y.-g. K.; Newton M. D.; Isied S. S. Distance Dependence of Electron Transfer across Peptides with Different Secondary Structures: The Role of Peptide Energetics and Electronic Coupling. J. Am. Chem. Soc. 2003, 125, 3722–3732. 10.1021/ja020358q. 12656602

[ref46] Coropceanu V.; Chen X.-K.; Wang T.; Zheng Z.; Brédas J.-L. Charge-Transfer Electronic States in Organic Solar Cells. Nat. Rev. Mater. 2019, 4, 689–707. 10.1038/s41578-019-0137-9.

[ref47] DuBose J. T.; Kamat P. V. Energy Versus Electron Transfer: Managing Excited-State Interactions in Perovskite Nanocrystal-Molecular Hybrids: Focus Review. Chem. Rev. 2022, 122, 12475–12494. 10.1021/acs.chemrev.2c00172. 35793168

[ref48] Uoyama H.; Goushi K.; Shizu K.; Nomura H.; Adachi C. Highly Efficient Organic Light-Emitting Diodes from Delayed Fluorescence. Nature 2012, 492, 234–238. 10.1038/nature11687. 23235877

[ref49] Berardi S.; La Ganga G.; Natali M.; Bazzan I.; Puntoriero F.; Sartorel A.; Scandola F.; Campagna S.; Bonchio M. Photocatalytic Water Oxidation: Tuning Light-Induced Electron Transfer by Molecular Co_4_O_4_ Cores. J. Am. Chem. Soc. 2012, 134, 11104–11107. 10.1021/ja303951z. 22716164

[ref50] Tay N. E.; Lehnherr D.; Rovis T. Photons or Electrons? A Critical Comparison of Electrochemistry and Photoredox Catalysis for Organic Synthesis. Chem. Rev. 2022, 122, 2487–2649. 10.1021/acs.chemrev.1c00384. 34751568 PMC10021920

[ref51] Tsai H.-S.; Shen C.-E.; Hsu S.-C.; Hsu L.-Y. Effects of Non-Adiabatic Electromagnetic Vacuum Fluctuations on Internal Conversion. J. Phys. Chem. Lett. 2023, 14, 5924–5931. 10.1021/acs.jpclett.3c01108. 37343274

[ref52] Frisch M. J.; Trucks G. W.; Schlegel H. B.; Scuseria G. E.; Robb M. A.; Cheeseman J. R.; Scalmani G.; Barone V.; Petersson G. A.; Nakatsuji H.; Gaussian 16, rev. C.01; Gaussian, Inc.: Wallingford, CT, 2016.

[ref53] Tomasi J.; Mennucci B.; Cammi R. Quantum Mechanical Continuum Solvation Models. Chem. Rev. 2005, 105, 2999–3094. 10.1021/cr9904009. 16092826

[ref54] Lu T.; Chen F. Multiwfn: A Multifunctional Wavefunction Analyzer. J. Comput. Chem. 2012, 33, 580–592. 10.1002/jcc.22885. 22162017

[ref55] Forde A.; Freixas V. M.; Fernandez-Alberti S.; Neukirch A. J.; Tretiak S. Charge-Transfer Luminescence in a Molecular Donor-Acceptor Complex: Computational Insights. J. Phys. Chem. Lett. 2022, 13, 8755–8760. 10.1021/acs.jpclett.2c02479. 36099248

[ref56] Lumerical, Inc. FDTD Solutions

[ref57] Haynes W. M. CRC Handbook of Chemistry and Physics; CRC Press: Boca Raton, FL, 2016.

[ref58] Johnson P. B.; Christy R.-W. Optical Constants of the Noble Metals. Phys. Rev. B 1972, 6, 4370 10.1103/PhysRevB.6.4370.

[ref59] Jordanides X. J.; Lang M. J.; Song X.; Fleming G. R. Solvation Dynamics in Protein Environments Studied by Photon Echo Spectroscopy. J. Phys. Chem. B 1999, 103, 7995–8005. 10.1021/jp9910993.

[ref60] Mertz E. L.; Krishtalik L. I. Low Dielectric Response in Enzyme Active Site. Proc. Natl. Acad. Sci. U.S.A. 2000, 97, 2081–2086. 10.1073/pnas.050316997. 10681440 PMC15757

