# Supplementary material for: Wide-Dynamic-Range Control of Quantum-Electrodynamic Electron Transfer Reactions in the Weak Coupling Regime
Source: J Phys Chem Lett. 2024 Jul 12;15(29):7403–10. doi: 10.1021/acs.jpclett.4c01265 (PMC11284844; doi:10.1021/acs.jpclett.4c01265)
Supplement: Supplementary file 1 — jz4c01265_si_001.pdf [file jz4c01265_si_001.pdf]

Supporting Information: Wide-Dynamic-Range Control of Quantum-Electrodynamic Electron Transfer Reactions in the Weak Coupling Regime

Yu-Chen Wei[*,†,‡,¶] and Liang-Yan Hsu[*,†,‡,§]

†*Institute of Atomic and Molecular Sciences, Academia Sinica, Taipei 106, Taiwan*

‡*Department of Chemistry, National Taiwan University, Taipei 106, Taiwan*

¶*Department of Applied Physics and Science Education, Eindhoven University of Technology, 5600MB Eindhoven, The Netherlands*

§*National Center for Theoretical Sciences, Taipei 106, Taiwan*

E-mail: y.c.wei@tue.nl; lyhsu@gate.sinica.edu.tw

Contents

S1 Model Hamiltonian **S3**

S2 Unitary Transformation along Polaritonic Coordinates **S6**

S3 Small-Polaron Transformation **S19**

S4 Derivation of Equations 9 and 10 **S20**

S4.1 Derivation of Marcus ET Rate S20

S4.2 Derivation of QED-Driven ET Rate S24

S5 Conversion of mQED-ET Theory to Cavity QED-ET Theory **S27**

S6 Conversion of mQED-ET Theory to Vacuum QED-ET Theory **S28**

S7 Supporting Figures **S29**

Supporting References **S38**

S1 Model Hamiltonian

According to the previous study,[1,2] the first two terms in Eq. 1 in the manuscript correspond to the standard model of molecular electron transfer (ET). As a result, these two terms can be simplified as a two-state molecular model in the electronic subspace with a harmonic vibrational bath. as shown in Eqs. S2–S4. In this framework, we regroup the terms in Eq. 1 in the manuscript as $\hat{H}_{\text{ele}}$—the electronic Hamiltonian, $\hat{H}_{\text{vib}}$—the vibrational Hamiltonian, $\hat{H}_{\text{ele-vib}}$—the electron-vibrational (vibronic) coupling, $\hat{H}_{\text{pol}}$—the polaritonic Hamiltonian and $\hat{H}_{\text{mol-pol}}$— the light-molecule interaction in the following,

$$\hat{H}_{\text{GM}} = \hat{H}_{\text{ele}} + \hat{H}_{\text{vib}} + \hat{H}_{\text{pol}} + \hat{H}_{\text{ele-vib}} + \hat{H}_{\text{mol-pol}}. \tag{S1}$$

$$\hat{H}_{\text{ele}} = E_{\text{DA}} \left|\text{DA}\right\rangle \left\langle\text{DA}\right| + E_{\text{D}^+\text{A}^-} \left|\text{D}^+\text{A}^-\right\rangle \left\langle\text{D}^+\text{A}^-\right| + V_{\text{ET}}(\left|\text{DA}\right\rangle \left\langle\text{D}^+\text{A}^-\right| + \text{h.c.}). \tag{S2}$$

$$\hat{H}_{\text{vib}} = \sum_{u=1}^{m_{\text{vib}}} \hbar\omega_{\text{vib},u} \hat{b}_u^\dagger \hat{b}_u. \tag{S3}$$

$$\hat{H}_{\text{ele-vib}} = \sum_{u=1}^{m_{\text{vib}}} \hbar\omega_{\text{vib},u} \left[\sqrt{S_{\text{vib},u}}(\hat{b}_u^\dagger + \hat{b}_u) + S_{\text{vib},u})\right] \left|\text{D}^+\text{A}^-\right\rangle \left\langle\text{D}^+\text{A}^-\right|. \tag{S4}$$

$$\hat{H}_{\text{pol}} = \int d\mathbf{r} \int_0^\infty d\omega \hbar\omega \hat{\mathbf{f}}^\dagger(\mathbf{r},\omega) \cdot \hat{\mathbf{f}}(\mathbf{r},\omega). \tag{S5}$$

$$\hat{H}_{\text{mol-pol}} = -\hat{\boldsymbol{\mu}} \cdot \hat{\mathbf{E}}(\mathbf{r}_{\text{M}}) + \frac{|\hat{\boldsymbol{\mu}}|^2}{2\epsilon_0 V_{\text{eff}}}. \tag{S6}$$

Here, the symbol D denotes electron-donor moiety, and the symbol A denotes electron-acceptor moiety; the symbol D^+ stands for the donor moiety losing an electron, and the symbol A^- stands for the acceptor moiety obtaining an electron. E_{DA} and $E_{\text{D}^+\text{A}^-}$ correspond to the energies of the initial and final states, respectively. V_{ET} is the Coulomb electronic coupling. The vibrational Hamiltonian $\hat{H}_{\text{vib}}$ is modeled as m_{vib} harmonic vibrational modes, and the electron-vibrational coupling $\hat{H}_{\text{ele-vib}}$ is modeled as a linear coupling between vibrational modes and the electronic state. The uth vibrational mode possesses the frequency $\omega_{\text{vib},u}$ with the vibrational creation (annihilation) operator $\hat{b}_u^\dagger(\hat{b}_u)$ and the vibrational Huang-Rhys factor $S_{\text{vib},u}$.

To evaluate ET rates, it is necessary to derive the matrix elements of the $\hat{H}_{\text{mol-pol}}$ in the electronic subspace. Within the Condon approximation, i.e., the nuclear degrees of freedom do not involve in light-molecule interaction, one can derive the permanent dipoles and the transition dipole as

$$\begin{aligned}
\boldsymbol{\mu}_{\text{DA}} &\equiv \langle \text{DA}| \hat{\boldsymbol{\mu}} |\text{DA}\rangle \,, \\
\boldsymbol{\mu}_{\text{D}^+\text{A}^-} &\equiv \langle \text{D}^+\text{A}^-| \hat{\boldsymbol{\mu}} |\text{D}^+\text{A}^-\rangle \,, \\
\boldsymbol{\mu}_{\text{DA},\text{D}^+\text{A}^-} &\equiv \langle \text{DA}| \hat{\boldsymbol{\mu}} |\text{D}^+\text{A}^-\rangle \\
\boldsymbol{\mu}_{\text{D}^+\text{A}^-,\text{DA}} &\equiv \langle \text{D}^+\text{A}^-| \hat{\boldsymbol{\mu}} |\text{DA}\rangle \,.
\end{aligned} \tag{S7}$$

To handle the linear term $\hat{\boldsymbol{\mu}} \cdot \hat{\mathbf{E}}(\mathbf{r}_{\text{M}})$ in Eq. 1 in the manuscript, we introduce the definition of electric field operator in the macroscipic QED,[3–5]

$$\hat{\mathbf{E}}(\mathbf{r}_{\text{M}}) \equiv \frac{i}{c^2}\sqrt{\frac{\hbar}{\pi\epsilon_0}} \int d\mathbf{r} \int_0^\infty d\omega \; \omega^2 \sqrt{\text{Im}\epsilon_{\text{r}}(\mathbf{r},\omega)} \; \overline{\overline{\mathbf{G}}}(\mathbf{r}_{\text{M}},\mathbf{r},\omega) \cdot \hat{\mathbf{f}}(\mathbf{r},\omega) + \text{h.c.}, \tag{S8}$$

where The tensor $\overline{\overline{\mathbf{G}}}(\mathbf{r}_{\text{M}},\mathbf{r},\omega)$ is the dyadic Green's function obeying the Maxwell equation $\left(\frac{\omega^2}{c^2}\epsilon_{\text{r}}(\mathbf{r}_{\text{M}},\omega) - \nabla\times\nabla\times\right)\overline{\overline{\mathbf{G}}}(\mathbf{r}_{\text{M}},\mathbf{r},\omega) = -\overline{\overline{\mathbf{I}}}_3\delta(\mathbf{r}_{\text{M}}-\mathbf{r})$. $\overline{\overline{\mathbf{I}}}_3$ is a 3×3 identity matrix and $\delta(\mathbf{r}_{\text{M}}-\mathbf{r})$ is the three-dimensional delta function.

Based on the definition of electric field operator Eq. S8 and Eq. S7, we define the polari-

tonic displacements as.

$$\mathbf{g}_{\mathrm{DA}}(\mathbf{r}_{\mathrm{M}},\mathbf{r},\omega)=\frac{i\omega}{c^2}\sqrt{\frac{\mathrm{Im}\epsilon_{\mathrm{r}}(\mathbf{r},\omega)}{\hbar\pi\epsilon_0}}\boldsymbol{\mu}_{\mathrm{DA}}\cdot\overline{\overline{\mathbf{G}}}(\mathbf{r}_{\mathrm{M}},\mathbf{r},\omega), \tag{S9}$$

$$\mathbf{g}_{\mathrm{D^+A^-}}(\mathbf{r}_{\mathrm{M}},\mathbf{r},\omega)=\frac{i\omega}{c^2}\sqrt{\frac{\mathrm{Im}\epsilon_{\mathrm{r}}(\mathbf{r},\omega)}{\hbar\pi\epsilon_0}}\boldsymbol{\mu}_{\mathrm{D^+A^-}}\cdot\overline{\overline{\mathbf{G}}}(\mathbf{r}_{\mathrm{M}},\mathbf{r},\omega), \tag{S10}$$

$$\mathbf{t}_{\mathrm{DA,D^+A^-}}(\mathbf{r}_{\mathrm{M}},\mathbf{r},\omega)=\frac{i\omega}{c^2}\sqrt{\frac{\mathrm{Im}\epsilon_{\mathrm{r}}(\mathbf{r},\omega)}{\hbar\pi\epsilon_0}}\boldsymbol{\mu}_{\mathrm{DA,D^+A^-}}\cdot\overline{\overline{\mathbf{G}}}(\mathbf{r}_{\mathrm{M}},\mathbf{r},\omega) \tag{S11}$$

$$\mathbf{t}_{\mathrm{D^+A^-,DA}}(\mathbf{r}_{\mathrm{M}},\mathbf{r},\omega)=\frac{i\omega}{c^2}\sqrt{\frac{\mathrm{Im}\epsilon_{\mathrm{r}}(\mathbf{r},\omega)}{\hbar\pi\epsilon_0}}\boldsymbol{\mu}_{\mathrm{D^+A^-,DA}}\cdot\overline{\overline{\mathbf{G}}}(\mathbf{r}_{\mathrm{M}},\mathbf{r},\omega). \tag{S12}$$

With Eqs. S9–S12, we can write down the matrix elements of $\hat{\boldsymbol{\mu}}\cdot\hat{\mathbf{E}}(\mathbf{r}_{\mathrm{M}})$

$$\begin{aligned}
&\hat{\boldsymbol{\mu}}\cdot\hat{\mathbf{E}}(\mathbf{r}_{\mathrm{M}})\\
&=\boldsymbol{\mu}_{\mathrm{DA}}\cdot\hat{\mathbf{E}}(\mathbf{r}_{\mathrm{M}})\left|\mathrm{DA}\right\rangle\left\langle\mathrm{DA}\right|+\boldsymbol{\mu}_{\mathrm{D^+A^-}}\cdot\hat{\mathbf{E}}(\mathbf{r}_{\mathrm{M}})\left|\mathrm{D^+A^-}\right\rangle\left\langle\mathrm{D^+A^-}\right|\\
&\quad+\boldsymbol{\mu}_{\mathrm{DA,D^+A^-}}\cdot\hat{\mathbf{E}}(\mathbf{r}_{\mathrm{M}})\left|\mathrm{DA}\right\rangle\left\langle\mathrm{D^+A^-}\right|+\boldsymbol{\mu}_{\mathrm{D^+A^-,DA}}\cdot\hat{\mathbf{E}}(\mathbf{r}_{\mathrm{M}})\left|\mathrm{D^+A^-}\right\rangle\left\langle\mathrm{DA}\right|\\
&=\int d\mathbf{r}\int_0^\infty d\omega\hbar\omega\Big(\mathbf{g}_{\mathrm{DA}}(\mathbf{r}_{\mathrm{M}},\mathbf{r},\omega)\cdot\hat{\mathbf{f}}(\mathbf{r},\omega)\left|\mathrm{DA}\right\rangle\left\langle\mathrm{DA}\right|+\mathbf{g}_{\mathrm{D^+A^-}}(\mathbf{r}_{\mathrm{M}},\mathbf{r},\omega)\cdot\hat{\mathbf{f}}(\mathbf{r},\omega)\left|\mathrm{D^+A^-}\right\rangle\left\langle\mathrm{D^+A^-}\right|\\
&\quad+\mathbf{t}_{\mathrm{DA,D^+A^-}}(\mathbf{r}_{\mathrm{M}},\mathbf{r},\omega)\cdot\hat{\mathbf{f}}(\mathbf{r},\omega)\left|\mathrm{DA}\right\rangle\left\langle\mathrm{D^+A^-}\right|+\mathbf{t}_{\mathrm{D^+A^-,DA}}(\mathbf{r}_{\mathrm{M}},\mathbf{r},\omega)\cdot\hat{\mathbf{f}}(\mathbf{r},\omega)\left|\mathrm{D^+A^-}\right\rangle\left\langle\mathrm{DA}\right|+\mathrm{h.c.}\Big).
\end{aligned} \tag{S13}$$

Similarly, the matrix elements of the dipole-self interaction in Eq. S6 take the form [1,6]

$$\begin{aligned}
&\frac{|\hat{\boldsymbol{\mu}}|^2}{2\epsilon_0V_{\mathrm{eff}}}\\
&=\frac{1}{2\epsilon_0V_{\mathrm{eff}}}\Big\{|\boldsymbol{\mu}_{\mathrm{DA}}|^2\left|\mathrm{DA}\right\rangle\left\langle\mathrm{DA}\right|+|\boldsymbol{\mu}_{\mathrm{D^+A^-}}|^2\left|\mathrm{D^+A^-}\right\rangle\left\langle\mathrm{D^+A^-}\right|\\
&+(\boldsymbol{\mu}_{\mathrm{DA}}+\boldsymbol{\mu}_{\mathrm{D^+A^-}})\cdot(\boldsymbol{\mu}_{\mathrm{DA,D^+A^-}}\left|\mathrm{DA}\right\rangle\left\langle\mathrm{D^+A^-}\right|+\boldsymbol{\mu}_{\mathrm{D^+A^-,DA}}\left|\mathrm{D^+A^-}\right\rangle\left\langle\mathrm{DA}\right|)+|\boldsymbol{\mu}_{\mathrm{DA,D^+A^-}}|^2I_2\Big\}.
\end{aligned} \tag{S14}$$

I_2 indicates the identity matrix $(\left|\mathrm{DA}\right\rangle\left\langle\mathrm{DA}\right|+\left|\mathrm{D^+A^-}\right\rangle\left\langle\mathrm{D^+A^-}\right|)$, which is neglected in the following derivation. Based on Eqs. S13 and S14, the matrix elements of the $\hat{H}_{\mathrm{mol-pol}}$ in the

electronic subspace can be expressed as

$$\begin{aligned}
&\hat{H}_{\text{mol-pol}} \\
&= -\int d\mathbf{r}\int_0^\infty d\omega\hbar\omega\Big(\mathbf{g}_{\text{DA}}(\mathbf{r}_{\text{M}},\mathbf{r},\omega)\cdot\hat{\mathbf{f}}(\mathbf{r},\omega)\left|\text{DA}\right\rangle\left\langle\text{DA}\right| + \mathbf{g}_{\text{D}^+\text{A}^-}(\mathbf{r}_{\text{M}},\mathbf{r},\omega)\cdot\hat{\mathbf{f}}(\mathbf{r},\omega)\left|\text{D}^+\text{A}^-\right\rangle\left\langle\text{D}^+\text{A}^-\right| \\
&\quad + \mathbf{t}_{\text{DA},\text{D}^+\text{A}^-}(\mathbf{r}_{\text{M}},\mathbf{r},\omega)\cdot\hat{\mathbf{f}}(\mathbf{r},\omega)\left|\text{DA}\right\rangle\left\langle\text{D}^+\text{A}^-\right| + \mathbf{t}_{\text{D}^+\text{A}^-,\text{DA}}(\mathbf{r}_{\text{M}},\mathbf{r},\omega)\cdot\hat{\mathbf{f}}(\mathbf{r},\omega)\left|\text{D}^+\text{A}^-\right\rangle\left\langle\text{DA}\right| + \text{h.c.}\Big) \\
&\quad + \frac{1}{2\epsilon_0 V_{\text{eff}}}\Big\{|\boldsymbol{\mu}_{\text{DA}}|^2\left|\text{DA}\right\rangle\left\langle\text{DA}\right| + |\boldsymbol{\mu}_{\text{D}^+\text{A}^-}|^2\left|\text{D}^+\text{A}^-\right\rangle\left\langle\text{D}^+\text{A}^-\right| \\
&\quad + (\boldsymbol{\mu}_{\text{DA}} + \boldsymbol{\mu}_{\text{D}^+\text{A}^-})\cdot(\boldsymbol{\mu}_{\text{DA},\text{D}^+\text{A}^-}\left|\text{DA}\right\rangle\left\langle\text{D}^+\text{A}^-\right| + \boldsymbol{\mu}_{\text{D}^+\text{A}^-,\text{DA}}\left|\text{D}^+\text{A}^-\right\rangle\left\langle\text{DA}\right|)\Big\}. \qquad \text{(S15)}
\end{aligned}$$

S2 Unitary Transformation along Polaritonic Coordinates

To cancel out the permanent dipole-field coupling in the electronic diagonal terms in Eq. (S15), we apply the unitary transformation along the polaritonic coordinates via[7]

$$\begin{aligned}
\hat{U}_{\text{pol}} \equiv \exp\Big\{\int d\mathbf{r}'\int_0^\infty d\omega'\big(&\mathbf{g}_{\text{DA}}(\mathbf{r}_{\text{M}},\mathbf{r}',\omega')\left|\text{DA}\right\rangle\left\langle\text{DA}\right| \\
&+ \mathbf{g}_{\text{D}^+\text{A}^-}(\mathbf{r}_{\text{M}},\mathbf{r}',\omega')\left|\text{D}^+\text{A}^-\right\rangle\left\langle\text{D}^+\text{A}^-\right|\big)\cdot\hat{\mathbf{f}}(\mathbf{r}',\omega') - \text{h.c.}\Big\}. \qquad \text{(S16)}
\end{aligned}$$

The transformed Hamiltonian can be expressed as

$$\hat{\mathcal{H}} \equiv \hat{U}_{\text{pol}}\hat{H}_{\text{GM}}\hat{U}_{\text{pol}}^\dagger = \hat{\mathcal{H}}_{\text{ele}} + \hat{\mathcal{H}}_{\text{vib}} + \hat{\mathcal{H}}_{\text{ele-vib}} + \hat{\mathcal{H}}_{\text{pol}} + \hat{\mathcal{H}}_{\text{mol-pol}} \qquad \text{(S17)}$$

The detailed derivation of Eq. S17 is shown below (Eqs. S18 − S40).

First, since $\hat{H}_{\text{vib}}$ and $\hat{H}_{\text{ele-vib}}$ commute with $\hat{U}_{\text{pol}}$, one can easily obtain the transformed Hamiltonian $\hat{\mathcal{H}}_{\text{vib}}$ and $\hat{\mathcal{H}}_{\text{ele-vib}}$, i.e.,

$$\hat{\mathcal{H}}_{\text{vib}} = \hat{H}_{\text{vib}}, \qquad \text{(S18)}$$

$$\hat{\mathcal{H}}_{\text{ele-vib}} = \hat{H}_{\text{ele-vib}}. \qquad \text{(S19)}$$

Next, the derivation of $\hat{\mathcal{H}}_{\rm ele}$ can be divided by two parts. First, the diagonal terms in $\hat{H}_{\rm ele}$ (Eq. S2) are unchanged after the unitary transformation. Second, the transformed off-diagonal terms in $\hat{H}_{\rm ele}$ (Eq. S2) can be simplified as

$$\begin{aligned}\hat{U}_{\rm pol}\left|{\rm DA}\right\rangle\left\langle{\rm D^{+}A^{-}}\right|\hat{U}_{\rm pol}^{\dagger} &= \exp\biggl\{\int d\mathbf{r}'\int_{0}^{\infty}d\omega'\mathbf{g}_{\rm DA}(\mathbf{r}_{\rm M},\mathbf{r}',\omega')\cdot\hat{\mathbf{f}}(\mathbf{r}',\omega')-{\rm h.c.}\biggr\}\left|{\rm DA}\right\rangle\left\langle{\rm D^{+}A^{-}}\right| \\ &\quad\times\exp\biggl\{-\int d\mathbf{r}'\int_{0}^{\infty}d\omega'\mathbf{g}_{\rm D^{+}A^{-}}(\mathbf{r}_{\rm M},\mathbf{r}',\omega')\cdot\hat{\mathbf{f}}(\mathbf{r}',\omega')+{\rm h.c.}\biggr\} \\ &= \exp(\hat{X}_{\rm DA})\left|{\rm DA}\right\rangle\left\langle{\rm D^{+}A^{-}}\right|\exp(-\hat{X}_{\rm D^{+}A^{-}}), \qquad \text{(S20)}\end{aligned}$$

where $\hat{X}_{\rm DA}$ and $\hat{X}_{\rm D^{+}A^{-}}$ are defined as

$$\begin{aligned}\hat{X}_{\rm DA} &= \int d\mathbf{r}'\int_{0}^{\infty}d\omega'\mathbf{g}_{\rm DA}(\mathbf{r}_{\rm M},\mathbf{r}',\omega')\cdot\hat{\mathbf{f}}(\mathbf{r}',\omega')-{\rm h.c.}, \\ \hat{X}_{\rm D^{+}A^{-}} &= \int d\mathbf{r}'\int_{0}^{\infty}d\omega'\mathbf{g}_{\rm D^{+}A^{-}}(\mathbf{r}_{\rm M},\mathbf{r}',\omega')\cdot\hat{\mathbf{f}}(\mathbf{r}',\omega')-{\rm h.c.}. \qquad \text{(S21)}\end{aligned}$$

In addition, we show that $\hat{X}_{\mathrm{DA}}$ and $\hat{X}_{\mathrm{D^+A^-}}$ commute.

$$
\begin{aligned}
&\big[\hat{X}_{\mathrm{DA}}, \hat{X}_{\mathrm{D^+A^-}}\big] \\
&= \int d\mathbf{r} \int d\mathbf{r}' \int_0^\infty d\omega \int_0^\infty d\omega' \\
&\quad \times \big[\mathbf{g}_{\mathrm{DA}}(\mathbf{r}_{\mathrm{M}}, \mathbf{r}, \omega) \cdot \hat{\mathbf{f}}(\mathbf{r}, \omega) - \text{h.c.}, \mathbf{g}_{\mathrm{D^+A^-}}(\mathbf{r}_{\mathrm{M}}, \mathbf{r}', \omega') \cdot \hat{\mathbf{f}}(\mathbf{r}', \omega') - \text{h.c.}\big] \\
&= \int d\mathbf{r} \int d\mathbf{r}' \int_0^\infty d\omega \int_0^\infty d\omega' \\
&\quad \times \Big\{ - \big[\mathbf{g}_{\mathrm{DA}}(\mathbf{r}_{\mathrm{M}}, \mathbf{r}, \omega) \cdot \hat{\mathbf{f}}(\mathbf{r}, \omega), \hat{\mathbf{f}}^\dagger(\mathbf{r}', \omega') \cdot \mathbf{g}^\dagger_{\mathrm{D^+A^-}}(\mathbf{r}_{\mathrm{M}}, \mathbf{r}', \omega')\big] \\
&\qquad - \big[\hat{\mathbf{f}}^\dagger(\mathbf{r}, \omega) \cdot \mathbf{g}^\dagger_{\mathrm{DA}}(\mathbf{r}_{\mathrm{M}}, \mathbf{r}, \omega), \mathbf{g}_{\mathrm{D^+A^-}}(\mathbf{r}_{\mathrm{M}}, \mathbf{r}', \omega') \cdot \hat{\mathbf{f}}(\mathbf{r}', \omega')\big] \Big\} \\
&= \int d\mathbf{r} \int d\mathbf{r}' \int_0^\infty d\omega \int_0^\infty d\omega' \\
&\quad \times \Big\{ \big[\hat{\mathbf{f}}^\dagger(\mathbf{r}', \omega') \cdot \mathbf{g}^\dagger_{\mathrm{D^+A^-}}(\mathbf{r}_{\mathrm{M}}, \mathbf{r}', \omega'), \mathbf{g}_{\mathrm{DA}}(\mathbf{r}_{\mathrm{M}}, \mathbf{r}, \omega) \cdot \hat{\mathbf{f}}(\mathbf{r}, \omega)\big] \\
&\qquad - \big[\hat{\mathbf{f}}^\dagger(\mathbf{r}, \omega) \cdot \mathbf{g}^\dagger_{\mathrm{DA}}(\mathbf{r}_{\mathrm{M}}, \mathbf{r}, \omega), \mathbf{g}_{\mathrm{D^+A^-}}(\mathbf{r}_{\mathrm{M}}, \mathbf{r}', \omega') \cdot \hat{\mathbf{f}}(\mathbf{r}', \omega')\big] \Big\} \\
&= \int d\mathbf{r} \int d\mathbf{r}' \int_0^\infty d\omega \int_0^\infty d\omega' \\
&\quad \times \Big\{ \big[\hat{\mathbf{f}}^\dagger(\mathbf{r}', \omega') \cdot \mathbf{g}^\dagger_{\mathrm{DA}}(\mathbf{r}_{\mathrm{M}}, \mathbf{r}', \omega'), \mathbf{g}_{\mathrm{D^+A^-}}(\mathbf{r}_{\mathrm{M}}, \mathbf{r}, \omega) \cdot \hat{\mathbf{f}}(\mathbf{r}, \omega)\big] \\
&\qquad - \big[\hat{\mathbf{f}}^\dagger(\mathbf{r}, \omega) \cdot \mathbf{g}^\dagger_{\mathrm{DA}}(\mathbf{r}_{\mathrm{M}}, \mathbf{r}, \omega), \mathbf{g}_{\mathrm{D^+A^-}}(\mathbf{r}_{\mathrm{M}}, \mathbf{r}', \omega') \cdot \hat{\mathbf{f}}(\mathbf{r}', \omega')\big] \Big\} \\
&= 0.
\end{aligned}
\tag{S22}
$$

As a result, we can use the relation[1]

$$
\exp(\hat{X})\exp(\hat{Y}) = \exp(\hat{X} + \hat{Y})\ \exp(\big[\hat{X}, \hat{Y}\big]/2), \tag{S23}
$$

which is valid if $\big[[\hat{Y},\hat{X}]\hat{X}\big]=0=\big[[\hat{Y},\hat{X}],\hat{Y}\big]$. Based on Eqs. (S21) and (S23), we can obtain

$$
\begin{aligned}
&\hat{U}_{\mathrm{pol}}\,|\mathrm{DA}\rangle\,\langle\mathrm{D^{+}A^{-}}|\,\hat{U}_{\mathrm{pol}}^{\dagger}\\
&=\exp(\hat{X}_{\mathrm{DA}})\,|\mathrm{DA}\rangle\,\langle\mathrm{D^{+}A^{-}}|\exp(-\hat{X}_{\mathrm{D^{+}A^{-}}})\\
&=|\mathrm{DA}\rangle\,\langle\mathrm{D^{+}A^{-}}|\exp(\hat{X}_{\mathrm{DA}}-\hat{X}_{\mathrm{D^{+}A^{-}}})\,\exp(-\frac{1}{2}\big[\hat{X}_{\mathrm{DA}},\hat{X}_{\mathrm{D^{+}A^{-}}}\big])\\
&=|\mathrm{DA}\rangle\,\langle\mathrm{D^{+}A^{-}}|\exp\Big\{\int d\mathbf{r}'\int_0^\infty d\omega'\big(\mathbf{g}_{\mathrm{DA}}(\mathbf{r}_{\mathrm{M}},\mathbf{r}',\omega')-\mathbf{g}_{\mathrm{D^{+}A^{-}}}(\mathbf{r}_{\mathrm{M}},\mathbf{r}',\omega')\big)\cdot\hat{\mathbf{f}}(\mathbf{r}',\omega')-\mathrm{h.c.}\Big\}\\
&=|\mathrm{DA}\rangle\,\langle\mathrm{D^{+}A^{-}}|\exp\Big\{-\int d\mathbf{r}'\int_0^\infty d\omega'\Delta\mathbf{g}(\mathbf{r}_{\mathrm{M}},\mathbf{r}',\omega')\cdot\hat{\mathbf{f}}(\mathbf{r}',\omega')+\mathrm{h.c.}\Big\}\\
&=|\mathrm{DA}\rangle\,\langle\mathrm{D^{+}A^{-}}|\,\hat{D}_{\mathrm{pol}},
\end{aligned}
\tag{S24}
$$

where $\Delta\mathbf{g}(\mathbf{r}_{\mathrm{M}},\mathbf{r}',\omega')\equiv\mathbf{g}_{\mathrm{D^{+}A^{-}}}(\mathbf{r}_{\mathrm{M}},\mathbf{r}',\omega')-\mathbf{g}_{\mathrm{DA}}(\mathbf{r}_{\mathrm{M}},\mathbf{r}',\omega')$ and $\hat{D}_{\mathrm{pol}}$ is the polaritonic displacement operator.

$$
\hat{D}_{\mathrm{pol}}\equiv\exp\Big\{-\int d\mathbf{r}'\int_0^\infty d\omega'\Delta\mathbf{g}(\mathbf{r}_{\mathrm{M}},\mathbf{r}',\omega')\cdot\hat{\mathbf{f}}(\mathbf{r}',\omega')+\mathrm{h.c.}\Big\}.
\tag{S25}
$$

According to Eq. S24, we can obtain $\hat{\mathcal{H}}_{\mathrm{ele}}$ as

$$
\hat{\mathcal{H}}_{\mathrm{ele}}=E_{\mathrm{DA}}\,|\mathrm{DA}\rangle\,\langle\mathrm{DA}|+E_{\mathrm{D^{+}A^{-}}}\,|\mathrm{D^{+}A^{-}}\rangle\,\langle\mathrm{D^{+}A^{-}}|+V_{\mathrm{ET}}(|\mathrm{DA}\rangle\,\langle\mathrm{D^{+}A^{-}}|\,\hat{D}_{\mathrm{pol}}+\mathrm{h.c.}).
\tag{S26}
$$

As for the derivation of $\hat{\mathcal{H}}_{\text{pol}}$ and $\hat{\mathcal{H}}_{\text{mol-pol}}$, we apply the relation $\exp(\hat{Y})\hat{X}\exp(-\hat{Y}) = \hat{X} + \left[\hat{Y}, \hat{X}\right]$, the commutator relation of bosonic vector field[3–5] and Eq. S16 to obtain

$$\begin{aligned}
&\hat{U}_{\text{pol}}\hat{\mathbf{f}}(\mathbf{r},\omega)\left|\text{DA}\right\rangle\left\langle\text{DA}\right|\hat{U}_{\text{pol}}^{\dagger} \\
&= \hat{\mathbf{f}}(\mathbf{r},\omega)\left|\text{DA}\right\rangle\left\langle\text{DA}\right| \\
&\quad + \left[\int d\mathbf{r}'\int_0^\infty d\omega'\big(\mathbf{g}_{\text{DA}}(\mathbf{r}_{\text{M}},\mathbf{r}',\omega')\cdot\hat{\mathbf{f}}(\mathbf{r}',\omega') - \text{h.c.}\big)\left|\text{DA}\right\rangle\left\langle\text{DA}\right|, \hat{\mathbf{f}}(\mathbf{r},\omega)\right] \\
&= \hat{\mathbf{f}}(\mathbf{r},\omega)\left|\text{DA}\right\rangle\left\langle\text{DA}\right| \\
&\quad - \left[\int d\mathbf{r}'\int_0^\infty d\omega'\hat{\mathbf{f}}^{\dagger}(\mathbf{r}',\omega')\cdot\mathbf{g}_{\text{DA}}^{\dagger}(\mathbf{r}_{\text{M}},\mathbf{r}',\omega')\left|\text{DA}\right\rangle\left\langle\text{DA}\right|, \hat{\mathbf{f}}(\mathbf{r},\omega)\right] \\
&= \hat{\mathbf{f}}(\mathbf{r},\omega)\left|\text{DA}\right\rangle\left\langle\text{DA}\right| \\
&\quad + \int d\mathbf{r}'\int_0^\infty d\omega'\delta(\mathbf{r}-\mathbf{r}')\delta(\omega-\omega')\mathbf{g}_{\text{DA}}^{\dagger}(\mathbf{r}_{\text{M}},\mathbf{r}',\omega')\left|\text{DA}\right\rangle\left\langle\text{DA}\right| \\
&= \big(\hat{\mathbf{f}}(\mathbf{r},\omega) + \mathbf{g}_{\text{DA}}^{\dagger}(\mathbf{r}_{\text{M}},\mathbf{r},\omega)\big)\left|\text{DA}\right\rangle\left\langle\text{DA}\right|. \qquad \text{(S27)}
\end{aligned}$$

Based on the similar approach,

$$\begin{aligned}
\hat{U}_{\text{pol}}\hat{\mathbf{f}}^{\dagger}(\mathbf{r},\omega)\left|\text{DA}\right\rangle\left\langle\text{DA}\right|\hat{U}_{\text{pol}}^{\dagger} &= \big(\hat{\mathbf{f}}^{\dagger}(\mathbf{r},\omega) + \mathbf{g}_{\text{DA}}(\mathbf{r}_{\text{M}},\mathbf{r},\omega)\big)\left|\text{DA}\right\rangle\left\langle\text{DA}\right|, \\
\hat{U}_{\text{pol}}\hat{\mathbf{f}}(\mathbf{r},\omega)\left|\text{D}^+\text{A}^-\right\rangle\left\langle\text{D}^+\text{A}^-\right|\hat{U}_{\text{pol}}^{\dagger} &= \big(\hat{\mathbf{f}}(\mathbf{r},\omega) + \mathbf{g}_{\text{D}^+\text{A}^-}^{\dagger}(\mathbf{r}_{\text{M}},\mathbf{r},\omega)\big)\left|\text{D}^+\text{A}^-\right\rangle\left\langle\text{D}^+\text{A}^-\right|, \\
\hat{U}_{\text{pol}}\hat{\mathbf{f}}^{\dagger}(\mathbf{r},\omega)\left|\text{D}^+\text{A}^-\right\rangle\left\langle\text{D}^+\text{A}^-\right|\hat{U}_{\text{pol}}^{\dagger} &= \big(\hat{\mathbf{f}}^{\dagger}(\mathbf{r},\omega) + \mathbf{g}_{\text{D}^+\text{A}^-}(\mathbf{r}_{\text{M}},\mathbf{r},\omega)\big)\left|\text{D}^+\text{A}^-\right\rangle\left\langle\text{D}^+\text{A}^-\right|. \qquad \text{(S28)}
\end{aligned}$$

Through Eqs. S24 and S27, we have

$$
\begin{aligned}
&\hat{U}_{\text{pol}}\hat{\mathbf{f}}(\mathbf{r},\omega)\left|\text{DA}\right\rangle\left\langle\text{D}^{+}\text{A}^{-}\right|\hat{U}^{\dagger}_{\text{pol}}\\
&=\exp\bigg\{\int d\mathbf{r}'\int_{0}^{\infty}d\omega'\mathbf{g}_{\text{DA}}(\mathbf{r}_{\text{M}},\mathbf{r}',\omega')\cdot\hat{\mathbf{f}}(\mathbf{r}',\omega')\left|\text{DA}\right\rangle\left\langle\text{DA}\right|-\text{h.c.}\bigg\}\hat{\mathbf{f}}(\mathbf{r},\omega)\\
&\quad\times\exp\bigg\{-\int d\mathbf{r}'\int_{0}^{\infty}d\omega'\mathbf{g}_{\text{DA}}(\mathbf{r}_{\text{M}},\mathbf{r}',\omega')\cdot\hat{\mathbf{f}}(\mathbf{r}',\omega')\left|\text{DA}\right\rangle\left\langle\text{DA}\right|+\text{h.c.}\bigg\}\\
&\quad\times\exp\bigg\{\int d\mathbf{r}'\int_{0}^{\infty}d\omega'\mathbf{g}_{\text{DA}}(\mathbf{r}_{\text{M}},\mathbf{r}',\omega')\cdot\hat{\mathbf{f}}(\mathbf{r}',\omega')\left|\text{DA}\right\rangle\left\langle\text{DA}\right|-\text{h.c.}\bigg\}\left|\text{DA}\right\rangle\left\langle\text{D}^{+}\text{A}^{-}\right|\\
&\quad\times\exp\bigg\{-\int d\mathbf{r}'\int_{0}^{\infty}d\omega'\mathbf{g}_{\text{D}^{+}\text{A}^{-}}(\mathbf{r}_{\text{M}},\mathbf{r}',\omega')\cdot\hat{\mathbf{f}}(\mathbf{r}',\omega')+\text{h.c.}\bigg\}\\
&=\big(\hat{\mathbf{f}}(\mathbf{r},\omega)+\mathbf{g}^{\dagger}_{\text{DA}}(\mathbf{r}_{\text{M}},\mathbf{r},\omega)\big)\left|\text{DA}\right\rangle\left\langle\text{D}^{+}\text{A}^{-}\right|\hat{D}_{\text{pol}}.
\end{aligned}
\tag{S29}
$$

In the same way,

$$
\begin{aligned}
&\hat{U}_{\text{pol}}\hat{\mathbf{f}}^{\dagger}(\mathbf{r},\omega)\left|\text{DA}\right\rangle\left\langle\text{D}^{+}\text{A}^{-}\right|\hat{U}^{\dagger}_{\text{pol}}=\big(\hat{\mathbf{f}}^{\dagger}(\mathbf{r},\omega)+\mathbf{g}_{\text{DA}}(\mathbf{r}_{\text{M}},\mathbf{r},\omega)\big)\left|\text{DA}\right\rangle\left\langle\text{D}^{+}\text{A}^{-}\right|\hat{D}_{\text{pol}},\\
&\hat{U}_{\text{pol}}\hat{\mathbf{f}}(\mathbf{r},\omega)\left|\text{D}^{+}\text{A}^{-}\right\rangle\left\langle\text{DA}\right|\hat{U}^{\dagger}_{\text{ph}}=\big(\hat{\mathbf{f}}(\mathbf{r},\omega)+\mathbf{g}^{\dagger}_{\text{DA}}(\mathbf{r}_{\text{M}},\mathbf{r},\omega)\big)\hat{D}^{\dagger}_{\text{pol}}\left|\text{D}^{+}\text{A}^{-}\right\rangle\left\langle\text{DA}\right|,\\
&\hat{U}_{\text{pol}}\hat{\mathbf{f}}^{\dagger}(\mathbf{r},\omega)\left|\text{D}^{+}\text{A}^{-}\right\rangle\left\langle\text{DA}\right|\hat{U}^{\dagger}_{\text{pol}}=\big(\hat{\mathbf{f}}^{\dagger}(\mathbf{r},\omega)+\mathbf{g}_{\text{DA}}(\mathbf{r}_{\text{M}},\mathbf{r},\omega)\big)\hat{D}^{\dagger}_{\text{pol}}\left|\text{D}^{+}\text{A}^{-}\right\rangle\left\langle\text{DA}\right|.
\end{aligned}
\tag{S30}
$$

By using Eqs. S27 and S28, we express the transformed diagonal terms of the electronic subspace in $\hat{H}_{\text{pol}} + \hat{H}_{\text{mol-pol}}$ (Eqs. S5 and S15) as

$$
\begin{aligned}
&\hat{U}_{\text{pol}} \int d\mathbf{r} \int_0^\infty d\omega\ \hbar\omega \Big\{ \hat{\mathbf{f}}^\dagger(\mathbf{r},\omega) \cdot \hat{\mathbf{f}}(\mathbf{r},\omega) \\
&- \Big(\mathbf{g}_{\text{DA}}(\mathbf{r}_{\text{M}},\mathbf{r},\omega) \cdot \hat{\mathbf{f}}(\mathbf{r},\omega) \left|\text{DA}\right\rangle \left\langle\text{DA}\right| + \mathbf{g}_{\text{D}^+\text{A}^-}(\mathbf{r}_{\text{M}},\mathbf{r},\omega) \cdot \hat{\mathbf{f}}(\mathbf{r},\omega) \left|\text{D}^+\text{A}^-\right\rangle \left\langle\text{D}^+\text{A}^-\right| + \text{h.c.} \Big) \Big\} \hat{U}_{\text{pol}}^\dagger, \\
&+ \hat{U}_{\text{pol}} \frac{1}{2\epsilon_0 V_{\text{eff}}} \big(|\boldsymbol{\mu}_{\text{DA}}|^2 \left|\text{DA}\right\rangle \left\langle\text{DA}\right| + |\boldsymbol{\mu}_{\text{D}^+\text{A}^-}|^2 \left|\text{D}^+\text{A}^-\right\rangle \left\langle\text{D}^+\text{A}^-\right| \big) \hat{U}_{\text{pol}}^\dagger \\
&= \int d\mathbf{r} \int_0^\infty d\omega\ \hbar\omega \Big\{ \hat{U}_{\text{pol}} \hat{\mathbf{f}}^\dagger(\mathbf{r},\omega) \hat{U}_{\text{pol}}^\dagger \cdot \hat{U}_{\text{pol}} \hat{\mathbf{f}}(\mathbf{r},\omega) \hat{U}_{\text{pol}}^\dagger \\
&\quad - \Big(\mathbf{g}_{\text{DA}}(\mathbf{r}_{\text{M}},\mathbf{r},\omega) \cdot \hat{U}_{\text{pol}} \hat{\mathbf{f}}(\mathbf{r},\omega) \hat{U}_{\text{pol}}^\dagger \left|\text{DA}\right\rangle \left\langle\text{DA}\right| \\
&\quad + \mathbf{g}_{\text{D}^+\text{A}^-}(\mathbf{r}_{\text{M}},\mathbf{r},\omega) \cdot \hat{U}_{\text{pol}} \hat{\mathbf{f}}(\mathbf{r},\omega) \hat{U}_{\text{pol}}^\dagger \left|\text{D}^+\text{A}^-\right\rangle \left\langle\text{D}^+\text{A}^-\right| + \text{h.c.} \Big) \Big\} \\
&\quad + \frac{1}{2\epsilon_0 V_{\text{eff}}} \big(|\boldsymbol{\mu}_{\text{DA}}|^2 \left|\text{DA}\right\rangle \left\langle\text{DA}\right| + |\boldsymbol{\mu}_{\text{D}^+\text{A}^-}|^2 \left|\text{D}^+\text{A}^-\right\rangle \left\langle\text{D}^+\text{A}^-\right| \big) \\
&= \int d\mathbf{r} \int_0^\infty d\omega\ \hbar\omega \Big\{ \hat{\mathbf{f}}^\dagger(\mathbf{r},\omega) \cdot \hat{\mathbf{f}}(\mathbf{r},\omega) \\
&\quad + \Big(\mathbf{g}_{\text{DA}}(\mathbf{r}_{\text{M}},\mathbf{r},\omega) \cdot \hat{\mathbf{f}}(\mathbf{r},\omega) \left|\text{DA}\right\rangle \left\langle\text{DA}\right| + \mathbf{g}_{\text{D}^+\text{A}^-}(\mathbf{r}_{\text{M}},\mathbf{r},\omega) \cdot \hat{\mathbf{f}}(\mathbf{r},\omega) \left|\text{D}^+\text{A}^-\right\rangle \left\langle\text{D}^+\text{A}^-\right| + \text{h.c.} \Big) \\
&\quad + \Big(|\mathbf{g}_{\text{DA}}(\mathbf{r}_{\text{M}},\mathbf{r},\omega)|^2 \left|\text{DA}\right\rangle \left\langle\text{DA}\right| + |\mathbf{g}_{\text{D}^+\text{A}^-}(\mathbf{r}_{\text{M}},\mathbf{r},\omega)|^2 \left|\text{D}^+\text{A}^-\right\rangle \left\langle\text{D}^+\text{A}^-\right| \Big) \\
&\quad - \Big(\mathbf{g}_{\text{DA}}(\mathbf{r}_{\text{M}},\mathbf{r},\omega) \cdot \hat{\mathbf{f}}(\mathbf{r},\omega) \left|\text{DA}\right\rangle \left\langle\text{DA}\right| + \mathbf{g}_{\text{D}^+\text{A}^-}(\mathbf{r}_{\text{M}},\mathbf{r},\omega) \cdot \hat{\mathbf{f}}(\mathbf{r},\omega) \left|\text{D}^+\text{A}^-\right\rangle \left\langle\text{D}^+\text{A}^-\right| + \text{h.c.} \Big) \\
&\quad - 2\Big(|\mathbf{g}_{\text{DA}}(\mathbf{r}_{\text{M}},\mathbf{r},\omega)|^2 \left|\text{DA}\right\rangle \left\langle\text{DA}\right| + |\mathbf{g}_{\text{D}^+\text{A}^-}(\mathbf{r}_{\text{M}},\mathbf{r},\omega)|^2 \left|\text{D}^+\text{A}^-\right\rangle \left\langle\text{D}^+\text{A}^-\right| \Big) \Big\} \\
&\quad + \frac{1}{2\epsilon_0 V_{\text{eff}}} \big(|\boldsymbol{\mu}_{\text{DA}}|^2 \left|\text{DA}\right\rangle \left\langle\text{DA}\right| + |\boldsymbol{\mu}_{\text{D}^+\text{A}^-}|^2 \left|\text{D}^+\text{A}^-\right\rangle \left\langle\text{D}^+\text{A}^-\right| \big) \\
&= \int d\mathbf{r} \int_0^\infty d\omega\ \hbar\omega \hat{\mathbf{f}}^\dagger(\mathbf{r},\omega) \cdot \hat{\mathbf{f}}(\mathbf{r},\omega) \\
&\quad - \int d\mathbf{r} \int_0^\infty d\omega\ \hbar\omega \big(|\mathbf{g}_{\text{DA}}(\mathbf{r}_{\text{M}},\mathbf{r},\omega)|^2 \left|\text{DA}\right\rangle \left\langle\text{DA}\right| + |\mathbf{g}_{\text{D}^+\text{A}^-}(\mathbf{r}_{\text{M}},\mathbf{r},\omega)|^2 \left|\text{D}^+\text{A}^-\right\rangle \left\langle\text{D}^+\text{A}^-\right| \big) \\
&\quad + \frac{1}{2\epsilon_0 V_{\text{eff}}} \big(|\boldsymbol{\mu}_{\text{DA}}|^2 \left|\text{DA}\right\rangle \left\langle\text{DA}\right| + |\boldsymbol{\mu}_{\text{D}^+\text{A}^-}|^2 \left|\text{D}^+\text{A}^-\right\rangle \left\langle\text{D}^+\text{A}^-\right| \big).
\end{aligned} \tag{S31}
$$

To reduce the quadratic terms in Eq. S31, we introduce the relations of dyadic Green's function.[5]

$$\mathrm{Im}\overline{\overline{\mathbf{G}}}(\mathbf{r}_\alpha, \mathbf{r}_\beta, \omega) = \int d\mathbf{r}\frac{\omega^2}{c^2}\mathrm{Im}\epsilon_\mathrm{r}(\mathbf{r}, \omega)\ \overline{\overline{\mathbf{G}}}(\mathbf{r}_\alpha, \mathbf{r}, \omega) \cdot \overline{\overline{\mathbf{G}}}^*(\mathbf{r}, \mathbf{r}_\beta, \omega), \tag{S32}$$

$$\int_0^\infty d\omega\frac{\omega}{c^2}\mathrm{Im}\overline{\overline{\mathbf{G}}}(\mathbf{r}_\alpha, \mathbf{r}_\beta, \omega) = \frac{\pi}{2}\ \overline{\overline{\mathbf{I}}}_3\delta(\mathbf{r}_\alpha - \mathbf{r}_\beta). \tag{S33}$$

Since $\delta(\mathbf{r}_\alpha - \mathbf{r}_\beta)$ is associated with the effective volume,[7] Eq. S33 can be expressed as

$$\begin{aligned}&\int_0^\infty d\omega\frac{\omega}{c^2}\mathrm{Im}\overline{\overline{\mathbf{G}}}(\mathbf{r}_\mathrm{M}, \mathbf{r}_\mathrm{M}, \omega)\\ &= \frac{\pi}{2V_\mathrm{eff}},\end{aligned} \tag{S34}$$

Based on the definition of $\mathbf{g}_{\mathrm{DA}}(\mathbf{r}_{\mathrm{M}}, \mathbf{r}, \omega)$ and $\mathbf{g}_{\mathrm{D^+A^-}}(\mathbf{r}_{\mathrm{M}}, \mathbf{r}, \omega)$ (Eqs. S9, S10), Eqs. S32 and S34, Eq. S31 can be reduced as

$$
\begin{aligned}
&\int d\mathbf{r} \int_0^\infty d\omega\ \hbar\omega \hat{\mathbf{f}}^\dagger(\mathbf{r}, \omega) \cdot \hat{\mathbf{f}}(\mathbf{r}, \omega) \\
&- \int d\mathbf{r} \int_0^\infty d\omega\ \hbar\omega \big(|\mathbf{g}_{\mathrm{DA}}(\mathbf{r}_{\mathrm{M}}, \mathbf{r}, \omega)|^2 \left|\mathrm{DA}\right\rangle \left\langle\mathrm{DA}\right| + |\mathbf{g}_{\mathrm{D^+A^-}}(\mathbf{r}_{\mathrm{M}}, \mathbf{r}, \omega)|^2 \left|\mathrm{D^+A^-}\right\rangle \left\langle\mathrm{D^+A^-}\right| \big) \\
&+ \frac{1}{2\epsilon_0 V_{\mathrm{eff}}} \big(|\boldsymbol{\mu}_{\mathrm{DA}}|^2 \left|\mathrm{DA}\right\rangle \left\langle\mathrm{DA}\right| + |\boldsymbol{\mu}_{\mathrm{D^+A^-}}|^2 \left|\mathrm{D^+A^-}\right\rangle \left\langle\mathrm{D^+A^-}\right| \big) \\
&= \int d\mathbf{r} \int_0^\infty d\omega\ \hbar\omega \hat{\mathbf{f}}^\dagger(\mathbf{r}, \omega) \cdot \hat{\mathbf{f}}(\mathbf{r}, \omega) \\
&\quad - \int d\mathbf{r} \int_0^\infty d\omega\ \frac{\omega^3}{c^4 \pi \epsilon_0} \mathrm{Im}\epsilon_{\mathrm{r}}(\mathbf{r}, \omega) \big(|\boldsymbol{\mu}_{\mathrm{DA}} \cdot \overline{\overline{\mathbf{G}}}(\mathbf{r}_{\mathrm{M}}, \mathbf{r}, \omega)|^2 \left|\mathrm{DA}\right\rangle \left\langle\mathrm{DA}\right| \\
&\qquad\qquad\qquad + |\boldsymbol{\mu}_{\mathrm{D^+A^-}} \cdot \overline{\overline{\mathbf{G}}}(\mathbf{r}_{\mathrm{M}}, \mathbf{r}, \omega)|^2 \left|\mathrm{D^+A^-}\right\rangle \left\langle\mathrm{D^+A^-}\right| \big) \\
&\quad + \frac{1}{2\epsilon_0 V_{\mathrm{eff}}} \big(|\boldsymbol{\mu}_{\mathrm{DA}}|^2 \left|\mathrm{DA}\right\rangle \left\langle\mathrm{DA}\right| + |\boldsymbol{\mu}_{\mathrm{D^+A^-}}|^2 \left|\mathrm{D^+A^-}\right\rangle \left\langle\mathrm{D^+A^-}\right| \big) \\
&= \int d\mathbf{r} \int_0^\infty d\omega\ \hbar\omega \hat{\mathbf{f}}^\dagger(\mathbf{r}, \omega) \cdot \hat{\mathbf{f}}(\mathbf{r}, \omega) \\
&\quad - \int_0^\infty d\omega\ \frac{\omega}{c^2 \pi \epsilon_0} \big(\boldsymbol{\mu}_{\mathrm{DA}} \cdot \mathrm{Im}\overline{\overline{\mathbf{G}}}(\mathbf{r}_{\mathrm{M}}, \mathbf{r}_{\mathrm{M}}, \omega) \cdot \boldsymbol{\mu}_{\mathrm{DA}} \left|\mathrm{DA}\right\rangle \left\langle\mathrm{DA}\right| \\
&\qquad\qquad\qquad + \boldsymbol{\mu}_{\mathrm{D^+A^-}} \cdot \mathrm{Im}\overline{\overline{\mathbf{G}}}(\mathbf{r}_{\mathrm{M}}, \mathbf{r}_{\mathrm{M}}, \omega) \cdot \boldsymbol{\mu}_{\mathrm{D^+A^-}} \left|\mathrm{D^+A^-}\right\rangle \left\langle\mathrm{D^+A^-}\right| \big) \\
&\quad + \frac{1}{2\epsilon_0 V_{\mathrm{eff}}} \big(|\boldsymbol{\mu}_{\mathrm{DA}}|^2 \left|\mathrm{DA}\right\rangle \left\langle\mathrm{DA}\right| + |\boldsymbol{\mu}_{\mathrm{D^+A^-}}|^2 \left|\mathrm{D^+A^-}\right\rangle \left\langle\mathrm{D^+A^-}\right| \big) \\
&= \int d\mathbf{r} \int_0^\infty d\omega\ \hbar\omega \hat{\mathbf{f}}^\dagger(\mathbf{r}, \omega) \cdot \hat{\mathbf{f}}(\mathbf{r}, \omega) \\
&\quad - \frac{1}{2\epsilon_0 V_{\mathrm{eff}}} \big(|\boldsymbol{\mu}_{\mathrm{DA}}|^2 \left|\mathrm{DA}\right\rangle \left\langle\mathrm{DA}\right| + |\boldsymbol{\mu}_{\mathrm{D^+A^-}}|^2 \left|\mathrm{D^+A^-}\right\rangle \left\langle\mathrm{D^+A^-}\right| \big) \\
&\quad + \frac{1}{2\epsilon_0 V_{\mathrm{eff}}} \big(|\boldsymbol{\mu}_{\mathrm{DA}}|^2 \left|\mathrm{DA}\right\rangle \left\langle\mathrm{DA}\right| + |\boldsymbol{\mu}_{\mathrm{D^+A^-}}|^2 \left|\mathrm{D^+A^-}\right\rangle \left\langle\mathrm{D^+A^-}\right| \big) \\
&= \int d\mathbf{r} \int_0^\infty d\omega\ \hbar\omega \hat{\mathbf{f}}^\dagger(\mathbf{r}, \omega) \cdot \hat{\mathbf{f}}(\mathbf{r}, \omega).
\end{aligned}
\tag{S35}
$$

In addition, by using Eqs. S24, S29, S30, the transformed off-diagonal terms of the electronic subspace in $\hat{H}_{\text{mol-pol}}$ (Eq. S15) can be organized as

$$
\begin{aligned}
&- \hat{U}_{\text{pol}} \int d\mathbf{r} \int_0^\infty d\omega \hbar\omega \bigg(\mathbf{t}_{\text{DA},\text{D}^+\text{A}^-}(\mathbf{r}_\text{M}, \mathbf{r}, \omega) \cdot \hat{\mathbf{f}}(\mathbf{r}, \omega) \left|\text{DA}\right\rangle \left\langle \text{D}^+\text{A}^-\right| \\
&\qquad\qquad + \mathbf{t}_{\text{D}^+\text{A}^-,\text{DA}}(\mathbf{r}_\text{M}, \mathbf{r}, \omega) \cdot \hat{\mathbf{f}}(\mathbf{r}, \omega) \left|\text{D}^+\text{A}^-\right\rangle \left\langle \text{DA}\right| + \text{h.c.} \bigg) \hat{U}^\dagger_{\text{pol}} \\
&+ \hat{U}_{\text{pol}} \frac{1}{2\epsilon_0 V_{\text{eff}}} (\boldsymbol{\mu}_{\text{DA}} + \boldsymbol{\mu}_{\text{D}^+\text{A}^-}) \cdot (\boldsymbol{\mu}_{\text{DA},\text{D}^+\text{A}^-} \left|\text{DA}\right\rangle \left\langle \text{D}^+\text{A}^-\right| + \boldsymbol{\mu}_{\text{D}^+\text{A}^-,\text{DA}} \left|\text{D}^+\text{A}^-\right\rangle \left\langle \text{DA}\right|) \hat{U}^\dagger_{\text{pol}} \\
&= -\hat{U}_{\text{pol}} \int d\mathbf{r} \int_0^\infty d\omega \hbar\omega \bigg(\mathbf{t}_{\text{DA},\text{D}^+\text{A}^-}(\mathbf{r}_\text{M}, \mathbf{r}, \omega) \cdot \hat{\mathbf{f}}(\mathbf{r}, \omega) \left|\text{DA}\right\rangle \left\langle \text{D}^+\text{A}^-\right| \\
&\qquad\qquad + \mathbf{t}_{\text{D}^+\text{A}^-,\text{DA}}(\mathbf{r}_\text{M}, \mathbf{r}, \omega) \cdot \hat{\mathbf{f}}(\mathbf{r}, \omega) \left|\text{D}^+\text{A}^-\right\rangle \left\langle \text{DA}\right| + \text{h.c.} \bigg) \hat{U}^\dagger_{\text{pol}} \\
&\quad + \frac{1}{2\epsilon_0 V_{\text{eff}}} (\boldsymbol{\mu}_{\text{DA}} + \boldsymbol{\mu}_{\text{D}^+\text{A}^-}) \cdot (\boldsymbol{\mu}_{\text{DA},\text{D}^+\text{A}^-} \left|\text{DA}\right\rangle \left\langle \text{D}^+\text{A}^-\right| \hat{D}_{\text{pol}} + \boldsymbol{\mu}_{\text{D}^+\text{A}^-,\text{DA}} \hat{D}^\dagger_{\text{pol}} \left|\text{D}^+\text{A}^-\right\rangle \left\langle \text{DA}\right|) \\
&= - \int d\mathbf{r} \int_0^\infty d\omega \hbar\omega \bigg(\mathbf{t}_{\text{DA},\text{D}^+\text{A}^-}(\mathbf{r}_\text{M}, \mathbf{r}, \omega) \cdot \hat{\mathbf{f}}(\mathbf{r}, \omega) \left|\text{DA}\right\rangle \left\langle \text{D}^+\text{A}^-\right| \hat{D}_{\text{pol}} \\
&\qquad\qquad + \mathbf{t}_{\text{D}^+\text{A}^-,\text{DA}}(\mathbf{r}_\text{M}, \mathbf{r}, \omega) \cdot \hat{\mathbf{f}}(\mathbf{r}, \omega) \hat{D}^\dagger_{\text{pol}} \left|\text{D}^+\text{A}^-\right\rangle \left\langle \text{DA}\right| + \text{h.c.} \bigg) \\
&\quad - \int d\mathbf{r} \int_0^\infty d\omega \hbar\omega \bigg(\mathbf{t}_{\text{DA},\text{D}^+\text{A}^-}(\mathbf{r}_\text{M}, \mathbf{r}, \omega) \cdot \mathbf{g}^\dagger_{\text{DA}}(\mathbf{r}_\text{M}, \mathbf{r}, \omega) \left|\text{DA}\right\rangle \left\langle \text{D}^+\text{A}^-\right| \hat{D}_{\text{pol}} \\
&\qquad\qquad + \mathbf{t}_{\text{D}^+\text{A}^-,\text{DA}}(\mathbf{r}_\text{M}, \mathbf{r}, \omega) \cdot \mathbf{g}^\dagger_{\text{DA}}(\mathbf{r}_\text{M}, \mathbf{r}, \omega) \hat{D}^\dagger_{\text{pol}} \left|\text{D}^+\text{A}^-\right\rangle \left\langle \text{DA}\right| + \text{h.c.} \bigg) \\
&\quad + \frac{1}{2\epsilon_0 V_{\text{eff}}} (\boldsymbol{\mu}_{\text{DA}} + \boldsymbol{\mu}_{\text{D}^+\text{A}^-}) \cdot (\boldsymbol{\mu}_{\text{DA},\text{D}^+\text{A}^-} \left|\text{DA}\right\rangle \left\langle \text{D}^+\text{A}^-\right| \hat{D}_{\text{pol}} + \boldsymbol{\mu}_{\text{D}^+\text{A}^-,\text{DA}} \hat{D}^\dagger_{\text{pol}} \left|\text{D}^+\text{A}^-\right\rangle \left\langle \text{DA}\right|).
\end{aligned}
\tag{S36}
$$

According to Eq. S8, The first integral correspond to the light-matter coupling $\boldsymbol{\mu}_{\mathrm{DA,D^+A^-}} \cdot \hat{\mathbf{E}}(\mathbf{r}_{\mathrm{M}})$. As a result, Eq. S36 can be simplified as

$$
\begin{aligned}
&-\int d\mathbf{r}\int_0^\infty d\omega\hbar\omega\Big(\mathbf{t}_{\mathrm{DA,D^+A^-}}(\mathbf{r}_{\mathrm{M}},\mathbf{r},\omega)\cdot\hat{\mathbf{f}}(\mathbf{r},\omega)\left|\mathrm{DA}\right\rangle\left\langle\mathrm{D^+A^-}\right|\hat{D}_{\mathrm{pol}} \\
&\qquad\qquad + \mathbf{t}_{\mathrm{D^+A^-,DA}}(\mathbf{r}_{\mathrm{M}},\mathbf{r},\omega)\cdot\hat{\mathbf{f}}(\mathbf{r},\omega)\hat{D}^\dagger_{\mathrm{pol}}\left|\mathrm{D^+A^-}\right\rangle\left\langle\mathrm{DA}\right| + \mathrm{h.c.}\Big) \\
&\quad-\int d\mathbf{r}\int_0^\infty d\omega\hbar\omega\Big(\mathbf{t}_{\mathrm{DA,D^+A^-}}(\mathbf{r}_{\mathrm{M}},\mathbf{r},\omega)\cdot\mathbf{g}^\dagger_{\mathrm{DA}}(\mathbf{r}_{\mathrm{M}},\mathbf{r},\omega)\left|\mathrm{DA}\right\rangle\left\langle\mathrm{D^+A^-}\right|\hat{D}_{\mathrm{pol}} \\
&\qquad\qquad + \mathbf{t}_{\mathrm{D^+A^-,DA}}(\mathbf{r}_{\mathrm{M}},\mathbf{r},\omega)\cdot\mathbf{g}^\dagger_{\mathrm{DA}}(\mathbf{r}_{\mathrm{M}},\mathbf{r},\omega)\hat{D}^\dagger_{\mathrm{pol}}\left|\mathrm{D^+A^-}\right\rangle\left\langle\mathrm{DA}\right| + \mathrm{h.c.}\Big) \\
&\quad+\frac{1}{2\epsilon_0 V_{\mathrm{eff}}}(\boldsymbol{\mu}_{\mathrm{DA}}+\boldsymbol{\mu}_{\mathrm{D^+A^-}})\cdot(\boldsymbol{\mu}_{\mathrm{DA,D^+A^-}}\left|\mathrm{DA}\right\rangle\left\langle\mathrm{D^+A^-}\right|\hat{D}_{\mathrm{pol}}+\boldsymbol{\mu}_{\mathrm{D^+A^-,DA}}\hat{D}^\dagger_{\mathrm{pol}}\left|\mathrm{D^+A^-}\right\rangle\left\langle\mathrm{DA}\right|) \\
&=-\boldsymbol{\mu}_{\mathrm{DA,D^+A^-}}\cdot\hat{\mathbf{E}}(\mathbf{r}_{\mathrm{M}})\left|\mathrm{DA}\right\rangle\left\langle\mathrm{D^+A^-}\right|\hat{D}_{\mathrm{pol}}-\boldsymbol{\mu}_{\mathrm{D^+A^-,DA}}\cdot\hat{\mathbf{E}}(\mathbf{r}_{\mathrm{M}})\hat{D}^\dagger_{\mathrm{pol}}\left|\mathrm{D^+A^-}\right\rangle\left\langle\mathrm{DA}\right| \\
&\quad-\int d\mathbf{r}\int_0^\infty d\omega\hbar\omega\Big(\mathbf{t}_{\mathrm{DA,D^+A^-}}(\mathbf{r}_{\mathrm{M}},\mathbf{r},\omega)\cdot\mathbf{g}^\dagger_{\mathrm{DA}}(\mathbf{r}_{\mathrm{M}},\mathbf{r},\omega)\left|\mathrm{DA}\right\rangle\left\langle\mathrm{D^+A^-}\right|\hat{D}_{\mathrm{pol}} \\
&\qquad\qquad + \mathbf{t}_{\mathrm{D^+A^-,DA}}(\mathbf{r}_{\mathrm{M}},\mathbf{r},\omega)\cdot\mathbf{g}^\dagger_{\mathrm{DA}}(\mathbf{r}_{\mathrm{M}},\mathbf{r},\omega)\hat{D}^\dagger_{\mathrm{pol}}\left|\mathrm{D^+A^-}\right\rangle\left\langle\mathrm{DA}\right| + \mathrm{h.c.}\Big) \\
&\quad+\frac{1}{2\epsilon_0 V_{\mathrm{eff}}}(\boldsymbol{\mu}_{\mathrm{DA}}+\boldsymbol{\mu}_{\mathrm{D^+A^-}})\cdot(\boldsymbol{\mu}_{\mathrm{DA,D^+A^-}}\left|\mathrm{DA}\right\rangle\left\langle\mathrm{D^+A^-}\right|\hat{D}_{\mathrm{pol}}+\boldsymbol{\mu}_{\mathrm{D^+A^-,DA}}\hat{D}^\dagger_{\mathrm{pol}}\left|\mathrm{D^+A^-}\right\rangle\left\langle\mathrm{DA}\right|)
\end{aligned}
\tag{S37}
$$

Based on the similar approach in Eq. S35, the integral in Eq. S37 can be reduced as

$$
\begin{aligned}
&-\int d\mathbf{r}\int_0^\infty d\omega\hbar\omega\Big(\mathbf{t}_{\mathrm{DA,D^+A^-}}(\mathbf{r}_\mathrm{M},\mathbf{r},\omega)\cdot\mathbf{g}^\dagger_{\mathrm{DA}}(\mathbf{r}_\mathrm{M},\mathbf{r},\omega)\left|\mathrm{DA}\right\rangle\left\langle\mathrm{D^+A^-}\right|\hat{D}_\mathrm{pol}\\
&\qquad\qquad+\mathbf{t}_{\mathrm{D^+A^-,DA}}(\mathbf{r}_\mathrm{M},\mathbf{r},\omega)\cdot\mathbf{g}^\dagger_{\mathrm{DA}}(\mathbf{r}_\mathrm{M},\mathbf{r},\omega)\hat{D}^\dagger_\mathrm{pol}\left|\mathrm{D^+A^-}\right\rangle\left\langle\mathrm{DA}\right|+\mathrm{h.c.}\Big)\\
&=-\int d\mathbf{r}\int_0^\infty d\omega\ \frac{\omega^3}{c^4\pi\epsilon_0}\mathrm{Im}\epsilon_\mathrm{r}(\mathbf{r},\omega)\\
&\quad\times\Big(\boldsymbol{\mu}_{\mathrm{DA,D^+A^-}}\cdot\overline{\overline{\mathbf{G}}}(\mathbf{r}_\mathrm{M},\mathbf{r},\omega)\cdot\overline{\overline{\mathbf{G}}}^*(\mathbf{r}_\mathrm{M},\mathbf{r},\omega)\cdot\boldsymbol{\mu}_\mathrm{DA}\left|\mathrm{DA}\right\rangle\left\langle\mathrm{D^+A^-}\right|\hat{D}_\mathrm{pol}\\
&\qquad+\boldsymbol{\mu}_{\mathrm{D^+A^-,DA}}\cdot\overline{\overline{\mathbf{G}}}(\mathbf{r}_\mathrm{M},\mathbf{r},\omega)\cdot\overline{\overline{\mathbf{G}}}^*(\mathbf{r}_\mathrm{M},\mathbf{r},\omega)\cdot\boldsymbol{\mu}_\mathrm{DA}\hat{D}^\dagger_\mathrm{pol}\left|\mathrm{D^+A^-}\right\rangle\left\langle\mathrm{DA}\right|+\mathrm{h.c.}\Big)\\
&=-\int_0^\infty d\omega\ \frac{2\omega}{c^2\pi\epsilon_0}\Big(\boldsymbol{\mu}_{\mathrm{DA,D^+A^-}}\cdot\mathrm{Im}\overline{\overline{\mathbf{G}}}(\mathbf{r}_\mathrm{M},\mathbf{r}_\mathrm{M},\omega)\cdot\boldsymbol{\mu}_\mathrm{DA}\left|\mathrm{DA}\right\rangle\left\langle\mathrm{D^+A^-}\right|\hat{D}_\mathrm{pol}\\
&\qquad+\boldsymbol{\mu}_{\mathrm{D^+A^-,DA}}\cdot\mathrm{Im}\overline{\overline{\mathbf{G}}}(\mathbf{r}_\mathrm{M},\mathbf{r}_\mathrm{M},\omega)\cdot\boldsymbol{\mu}_\mathrm{DA}\hat{D}^\dagger_\mathrm{pol}\left|\mathrm{D^+A^-}\right\rangle\left\langle\mathrm{DA}\right|\Big)\\
&=-\frac{1}{\epsilon_0V_\mathrm{eff}}\Big(\boldsymbol{\mu}_{\mathrm{DA,D^+A^-}}\cdot\boldsymbol{\mu}_\mathrm{DA}\left|\mathrm{DA}\right\rangle\left\langle\mathrm{D^+A^-}\right|\hat{D}_\mathrm{pol}+\boldsymbol{\mu}_{\mathrm{D^+A^-,DA}}\cdot\boldsymbol{\mu}_\mathrm{DA}\hat{D}^\dagger_\mathrm{pol}\left|\mathrm{D^+A^-}\right\rangle\left\langle\mathrm{DA}\right|\Big)
\end{aligned}
\tag{S38}
$$

According to Eq. S38, Eq. S37 becomes

$$
\begin{aligned}
&-\boldsymbol{\mu}_{\mathrm{DA,D^+A^-}}\cdot\hat{\mathbf{E}}(\mathbf{r}_\mathrm{M})\left|\mathrm{DA}\right\rangle\left\langle\mathrm{D^+A^-}\right|\hat{D}_\mathrm{pol}-\boldsymbol{\mu}_{\mathrm{D^+A^-,DA}}\cdot\hat{\mathbf{E}}(\mathbf{r}_\mathrm{M})\hat{D}^\dagger_\mathrm{pol}\left|\mathrm{D^+A^-}\right\rangle\left\langle\mathrm{DA}\right|\\
&\quad-\int d\mathbf{r}\int_0^\infty d\omega\hbar\omega\Big(\mathbf{t}_{\mathrm{DA,D^+A^-}}(\mathbf{r}_\mathrm{M},\mathbf{r},\omega)\cdot\mathbf{g}^\dagger_{\mathrm{DA}}(\mathbf{r}_\mathrm{M},\mathbf{r},\omega)\left|\mathrm{DA}\right\rangle\left\langle\mathrm{D^+A^-}\right|\hat{D}_\mathrm{pol}\\
&\qquad\qquad+\mathbf{t}_{\mathrm{D^+A^-,DA}}(\mathbf{r}_\mathrm{M},\mathbf{r},\omega)\cdot\mathbf{g}^\dagger_{\mathrm{DA}}(\mathbf{r}_\mathrm{M},\mathbf{r},\omega)\hat{D}^\dagger_\mathrm{pol}\left|\mathrm{D^+A^-}\right\rangle\left\langle\mathrm{DA}\right|+\mathrm{h.c.}\Big)\\
&\quad+\frac{1}{2\epsilon_0V_\mathrm{eff}}(\boldsymbol{\mu}_\mathrm{DA}+\boldsymbol{\mu}_{\mathrm{D^+A^-}})\cdot(\boldsymbol{\mu}_{\mathrm{DA,D^+A^-}}\left|\mathrm{DA}\right\rangle\left\langle\mathrm{D^+A^-}\right|\hat{D}_\mathrm{pol}+\boldsymbol{\mu}_{\mathrm{D^+A^-,DA}}\hat{D}^\dagger_\mathrm{pol}\left|\mathrm{D^+A^-}\right\rangle\left\langle\mathrm{DA}\right|)\\
&=-\boldsymbol{\mu}_{\mathrm{DA,D^+A^-}}\cdot\hat{\mathbf{E}}(\mathbf{r}_\mathrm{M})\left|\mathrm{DA}\right\rangle\left\langle\mathrm{D^+A^-}\right|\hat{D}_\mathrm{pol}-\boldsymbol{\mu}_{\mathrm{D^+A^-,DA}}\cdot\hat{\mathbf{E}}(\mathbf{r}_\mathrm{M})\hat{D}^\dagger_\mathrm{pol}\left|\mathrm{D^+A^-}\right\rangle\left\langle\mathrm{DA}\right|\\
&\quad+\frac{1}{2\epsilon_0V_\mathrm{eff}}(\boldsymbol{\mu}_{\mathrm{D^+A^-}}-\boldsymbol{\mu}_\mathrm{DA})\cdot(\boldsymbol{\mu}_{\mathrm{DA,D^+A^-}}\left|\mathrm{DA}\right\rangle\left\langle\mathrm{D^+A^-}\right|\hat{D}_\mathrm{pol}+\boldsymbol{\mu}_{\mathrm{D^+A^-,DA}}\hat{D}^\dagger_\mathrm{pol}\left|\mathrm{D^+A^-}\right\rangle\left\langle\mathrm{DA}\right|)\\
&=(-\boldsymbol{\mu}_{\mathrm{DA,D^+A^-}}\cdot\hat{\mathbf{E}}(\mathbf{r}_\mathrm{M})+\frac{1}{2\epsilon_0V_\mathrm{eff}}\Delta\boldsymbol{\mu}\cdot\boldsymbol{\mu}_{\mathrm{DA,D^+A^-}})\left|\mathrm{DA}\right\rangle\left\langle\mathrm{D^+A^-}\right|\hat{D}_\mathrm{pol}+\mathrm{h.c.},
\end{aligned}
\tag{S39}
$$

where $\Delta\boldsymbol{\mu}\equiv\boldsymbol{\mu}_{\mathrm{D^+A^-}}-\boldsymbol{\mu}_\mathrm{DA}$.

On the basis of Eqs. S35 and S39, $\hat{\mathcal{H}}_{\text{pol}} + \hat{\mathcal{H}}_{\text{mol-pol}}$ is derived as

$$\begin{aligned}
&\hat{\mathcal{H}}_{\text{pol}} + \hat{\mathcal{H}}_{\text{mol-pol}} \\
&= \int d\mathbf{r} \int_0^\infty d\omega \ \hbar\omega \hat{\mathbf{f}}^\dagger(\mathbf{r},\omega) \cdot \hat{\mathbf{f}}(\mathbf{r},\omega) \\
&\quad + \left((-\boldsymbol{\mu}_{\text{DA,D}^+\text{A}^-} \cdot \hat{\mathbf{E}}(\mathbf{r}_\text{M}) + \frac{\Delta\boldsymbol{\mu} \cdot \boldsymbol{\mu}_{\text{DA,D}^+\text{A}^-}}{2\epsilon_0 V_{\text{eff}}}) |\text{DA}\rangle \langle \text{D}^+\text{A}^-| \hat{D}_{\text{pol}} + \text{h.c.} \right).
\end{aligned} \tag{S40}$$

According to our previous study,[7] the term $\frac{\Delta\boldsymbol{\mu} \cdot \boldsymbol{\mu}_{\text{DA,D}^+\text{A}^-}}{2\epsilon_0 V_{\text{eff}}}$ indicates the reorganization dipole self-coupling Λ_{pol}.

$$\Lambda_{\text{pol}} \equiv \frac{\Delta\boldsymbol{\mu} \cdot \boldsymbol{\mu}_{\text{DA,D}^+\text{A}^-}}{2\epsilon_0 V_{\text{eff}}}. \tag{S41}$$

In summary, the transformed Hamiltonian is shown as follows.

$$\begin{aligned}
\hat{\mathcal{H}} \ &\equiv \hat{U}_{\text{pol}} \hat{H}_{\text{GM}} \hat{U}_{\text{pol}}^\dagger = \hat{\mathcal{H}}_{\text{ele}} + \hat{\mathcal{H}}_{\text{vib}} + \hat{\mathcal{H}}_{\text{ele-vib}} + \hat{\mathcal{H}}_{\text{pol}} + \hat{\mathcal{H}}_{\text{mol-pol}}, \\
\hat{\mathcal{H}}_{\text{ele}} &= E_{\text{DA}} |\text{DA}\rangle \langle \text{DA}| + E_{\text{D}^+\text{A}^-} |\text{D}^+\text{A}^-\rangle \langle \text{D}^+\text{A}^-| + V_{\text{ET}}(|\text{DA}\rangle \langle \text{D}^+\text{A}^-| \hat{D}_{\text{pol}} + \text{h.c.}), \\
\hat{\mathcal{H}}_{\text{vib}} &= \hat{H}_{\text{vib}}, \\
\hat{\mathcal{H}}_{\text{ele-vib}} &= \hat{H}_{\text{ele-vib}}, \\
\hat{\mathcal{H}}_{\text{pol}} &= \int d\mathbf{r} \int_0^\infty d\omega \ \hbar\omega \hat{\mathbf{f}}^\dagger(\mathbf{r},\omega) \cdot \hat{\mathbf{f}}(\mathbf{r},\omega), \\
\hat{\mathcal{H}}_{\text{mol}-\text{pol}} &= (-\boldsymbol{\mu}_{\text{DA,D}^+\text{A}^-} \cdot \hat{\mathbf{E}}(\mathbf{r}_\text{M}) + \Lambda_{\text{pol}}) |\text{DA}\rangle \langle \text{D}^+\text{A}^-| \hat{D}_{\text{pol}} + \text{h.c.}.
\end{aligned} \tag{S42}$$

S3 Small-Polaron Transformation

Here we apply the unitary transformation along the vibrational basis via[8,9]

$$\hat{U}_{\mathrm{vib}} \equiv \exp\left\{ \sum_{u=1}^{m_{\mathrm{vib}}} \sqrt{S_{\mathrm{vib},u}}(\hat{b}_u^\dagger - \hat{b}_u)) \left|\mathrm{D^+A^-}\right\rangle \left\langle \mathrm{D^+A^-}\right| \right\}, \tag{S43}$$

The transformed Hamiltonian can be written as follows.

$$\hat{\mathscr{H}} \equiv \hat{U}_{\mathrm{vib}}\hat{\mathcal{H}}\hat{U}_{\mathrm{vib}}^\dagger = \hat{\mathscr{H}}_{\mathrm{ele}} + \hat{\mathscr{H}}_{\mathrm{vib}} + \hat{\mathscr{H}}_{\mathrm{pol}} + \hat{\mathscr{H}}_{\mathrm{mol-pol}}, \tag{S44}$$

$$\hat{\mathscr{H}}_{\mathrm{ele}} \approx E_{\mathrm{DA}} \left|\mathrm{DA}\right\rangle \left\langle \mathrm{DA}\right| + E_{\mathrm{D^+A^-}} \left|\mathrm{D^+A^-}\right\rangle \left\langle \mathrm{D^+A^-}\right| + V_{\mathrm{ET}}(\left|\mathrm{DA}\right\rangle \left\langle \mathrm{D^+A^-}\right| \hat{D}_{\mathrm{pol}}\hat{D}_{\mathrm{vib}} + \mathrm{h.c.}), \tag{S45}$$

$$\hat{\mathscr{H}}_{\mathrm{vib}} = \sum_{u=1}^{m_{\mathrm{vib}}} \hbar\omega_{\mathrm{vib},u}\hat{b}_u^\dagger\hat{b}_u, \tag{S46}$$

$$\hat{\mathscr{H}}_{\mathrm{pol}} = \int d\mathbf{r} \int_0^\infty d\omega\ \hbar\omega\hat{\mathbf{f}}^\dagger(\mathbf{r},\omega)\cdot\hat{\mathbf{f}}(\mathbf{r},\omega), \tag{S47}$$

$$\hat{\mathscr{H}}_{\mathrm{mol-pol}} = (-\boldsymbol{\mu}_{\mathrm{DA,D^+A^-}}\cdot\hat{\mathbf{E}}(\mathbf{r}_{\mathrm{M}}) + \Lambda_{\mathrm{pol}}) \left|\mathrm{DA}\right\rangle \left\langle \mathrm{D^+A^-}\right| \hat{D}_{\mathrm{pol}}\hat{D}_{\mathrm{vib}} + \mathrm{h.c.}, \tag{S48}$$

where $\hat{D}_{\mathrm{vib}} = \exp\left\{\sum_{u=1}^{m_{\mathrm{vib}}} \sqrt{S_{\mathrm{vib},u}}(\hat{b}_u^\dagger - \hat{b}_u)\right\}$. According to Eqs. S45 and S48, we can define the new effective electronic coupling $\hat{\mathscr{V}}_{\mathrm{ET}}$ by collecting all the electronic off-diagonal terms together.

$$\hat{\mathscr{V}}_{\mathrm{ET}} \equiv \left(\mathrm{V}_{\mathrm{ET}} - \boldsymbol{\mu}_{\mathrm{DA,D^+A^-}}\cdot\hat{\mathbf{E}}(\mathbf{r}_{\mathrm{M}}) + \Lambda_{\mathrm{pol}}\right) \left|\mathrm{DA}\right\rangle \left\langle \mathrm{D^+A^-}\right| \hat{D}_{\mathrm{pol}}\hat{D}_{\mathrm{vib}} + \mathrm{h.c.}. \tag{S49}$$

S4 Derivation of Equations 9 and 10

First, we denote $\{M\} \equiv \{M_1, \dots, M_u, \dots, M_{m_{\rm vib}}\}$ and $\{M'\}$ the initial and final states of the vibrational basis. Note that the notation $(')$ represents the basis of the final state. Based on this setting and the Fermi's golden rule, the total ET rate can be expressed as follows.

$$k_{\rm ET} = k_{\rm Marcus} + k_{\rm QED}, \tag{S50}$$

$$k_{\rm Marcus} = \frac{2\pi}{\hbar} \sum_{\{M\},\{M'\}} {\rm P}_{\{M\}} |\langle {\rm DA}, \{M\}, \{0\}| \hat{\mathscr{V}}_{\rm ET} |{\rm D^+A^-}, \{M'\}, \{0\}\rangle|^2 \times \delta(E_{{\rm DA},\{M\},\{0\}} - E_{{\rm D^+A^-},\{M'\},\{0\}}), \tag{S51}$$

$$\begin{aligned} k_{\rm QED} &= \int d\mathbf{r} \int_0^\infty d\omega \ \kappa_{{\rm ET},\{0\}\to\{\mathbf{1}(\mathbf{r},\omega)\}} \\ &= \frac{2\pi}{\hbar} \sum_{\{M\},\{M'\}} {\rm P}_{\{M\}} \int d\mathbf{r} \int_0^\infty d\omega \ |\langle {\rm DA}, \{M\}, \{0\}| \hat{\mathscr{V}}_{\rm ET} |{\rm D^+A^-}, \{M'\}, \{\mathbf{1}(\mathbf{r},\omega)\}\rangle|^2 \\ &\quad \times \delta(E_{{\rm DA},\{M\},\{0\}} - E_{{\rm D^+A^-},\{M'\},\{\mathbf{1}(\mathbf{r},\omega)\}}). \end{aligned} \tag{S52}$$

${\rm P}_{\{M\}}$ is the population distribution that characterizes the vibrational bath. $|{\rm D^+A^-}, \{M'\}, \{0\}\rangle = |{\rm D^+A^-}\rangle \otimes |\{M'\}\rangle \otimes \{0\}$ stands for a Fock state which describes the final electronic state $|{\rm D^+A^-}\rangle$ combined with the vibrational state $|\{M'\}\rangle$ and a polaritonic state $\{0\}$. Here, $\{0\}$ and $\{\mathbf{1}(\mathbf{r},\omega)\}$ indicate the vacuum polaritonic state and the single polaritonic state with the frequency ω monitored at the position $\mathbf{r}$, respectively. Note that we neglect the temperature effect on the photon population and assume that the initial population of photons is fully at the polariton vacuum state.[6,10]

S4.1 Derivation of Marcus ET Rate

According to Eq. S51, we can evaluate $k_{\rm Marcus}$ and obtain exactly the same formula derived from the famous Marcus theory.[2] By using the identity $\delta(x) = (2\pi\hbar)^{-1} \int_{-\infty}^{\infty} {\rm d}t \, \exp(ixt/\hbar)$ and a completeness relation $\sum_{\{M'\}} \left(|\{M'\}\rangle \langle \{M'\}| \right)$ in the vibrational subspace, we can

rewrite Eq. S51 in the form,

$$
\begin{aligned}
k_{\mathrm{Marcus}} &= \frac{1}{\hbar^2} \sum_{\{M\},\{M'\}} \mathrm{P}_{\{M\}} \langle \mathrm{DA}, \{M\}, \{0\}| \hat{\mathscr{V}}_{\mathrm{ET}} |\mathrm{D^{+}A^{-}}, \{M'\}, \{0\}\rangle \\
&\quad \times \langle \mathrm{D^{+}A^{-}}, \{M'\}, \{0\}| \hat{\mathscr{V}}_{\mathrm{ET}} |\mathrm{DA}, \{M\}, \{0\}\rangle \int_{-\infty}^{\infty} \mathrm{d}t \, \exp\left\{ \frac{i(E_{\mathrm{DA},\{M\},\{0\}} - E_{\mathrm{D^{+}A^{-}},\{M'\},\{0\}})t}{\hbar} \right\} \\
&= \frac{1}{\hbar^2} \sum_{\{M\}} \mathrm{P}_{\{M\}} \langle \{M\}| e^{\frac{i\hat{\mathscr{H}}_{\mathrm{vib}} t}{\hbar}} \hat{\mathscr{V}}^{\{0\},\{0\}}_{\mathrm{DA,D^{+}A^{-}}} e^{-\frac{i\hat{\mathscr{H}}_{\mathrm{vib}} t}{\hbar}} \\
&\quad \times \sum_{\{M'\}} \big(|\{M'\}\rangle \langle \{M'\}| \big) \hat{\mathscr{V}}^{\{0\},\{0\}}_{\mathrm{D^{+}A^{-},DA}} |\{M\}\rangle \int_{-\infty}^{\infty} \mathrm{d}t \, \exp\left\{ \frac{i(E_{\mathrm{DA},\{0\}} - E_{\mathrm{D^{+}A^{-}},\{0\}})t}{\hbar} \right\} \\
&= \frac{1}{\hbar^2} \int_{-\infty}^{\infty} \mathrm{d}t \, \exp\big\{ \frac{-i\Delta E t}{\hbar} \big\} \langle \hat{\mathscr{V}}^{\{0\},\{0\}}_{\mathrm{DA,D^{+}A^{-}}}(t) \hat{\mathscr{V}}^{\{0\},\{0\}}_{\mathrm{D^{+}A^{-},DA}} \rangle, \qquad \text{(S53)}
\end{aligned}
$$

where $\langle \cdots \rangle$ denotes a thermal average in the vibrational subspace and $\hat{\mathscr{V}}^{\{0\},\{0\}}_{\mathrm{DA,D^{+}A^{-}}}(t) = \exp\big\{\frac{i\hat{\mathscr{H}}_{\mathrm{vib}} t}{\hbar}\big\}$ $\hat{\mathscr{V}}^{\{0\},\{0\}}_{\mathrm{DA,D^{+}A^{-}}} \exp\big\{ - \frac{i\hat{\mathscr{H}}_{\mathrm{vib}} t}{\hbar}\big\}$. Note that $\hat{\mathscr{V}}^{\{0\},\{0\}}_{\mathrm{DA,D^{+}A^{-}}}$ is the electronic coupling projected on the electronic and photonic basis, and it can be expressed as

$$
\begin{aligned}
\hat{\mathscr{V}}^{\{0\},\{0\}}_{\mathrm{DA,D^{+}A^{-}}} &= (\hat{\mathscr{V}}^{\{0\},\{0\}}_{\mathrm{D^{+}A^{-},DA}})^{\dagger} \\
&= \langle \mathrm{DA}, \{0\}| \hat{\mathscr{V}}_{\mathrm{ET}} |\mathrm{D^{+}A^{-}}, \{0\}\rangle \\
&= \big((\mathrm{V_{ET}} + \Lambda_{\mathrm{pol}}) \langle \{0\}| \hat{D}_{\mathrm{pol}} |\{0\}\rangle - \langle \{0\}| \boldsymbol{\mu}_{\mathrm{DA,D^{+}A^{-}}} \cdot \hat{\mathbf{E}}(\mathbf{r}_{\mathrm{M}}) \hat{D}_{\mathrm{pol}} |\{0\}\rangle \big) \hat{D}_{\mathrm{vib}}. \qquad \text{(S54)}
\end{aligned}
$$

Due to the effects of polaritonic displacements, the second term of Eq. S54 is non-zero. According to the 0-0 polaritonic Franck-Condon overlap derived in our previous study[7] and the derivation in Eq. S38, this term becomes

$$\begin{aligned}
& - \langle\{0\}| \boldsymbol{\mu}_{\mathrm{DA,D^+A^-}} \cdot \hat{\mathbf{E}}(\mathbf{r}_{\mathrm{M}})\hat{D}_{\mathrm{pol}} |\{0\}\rangle \\
&= -\int d\mathbf{r}' \int_0^\infty d\omega' \hbar\omega' \big(\langle\{0\}| \mathbf{t}_{\mathrm{DA,D^+A^-}}(\mathbf{r}_{\mathrm{M}},\mathbf{r}',\omega') \cdot \hat{\mathbf{f}}(\mathbf{r}',\omega')\hat{D}_{\mathrm{pol}} |\{0\}\rangle \\
&\qquad\qquad + \langle\{0\}| \hat{\mathbf{f}}^\dagger(\mathbf{r}',\omega') \cdot \mathbf{t}^\dagger_{\mathrm{DA,D^+A^-}}(\mathbf{r}_{\mathrm{M}},\mathbf{r}',\omega')\hat{D}_{\mathrm{pol}} |\{0\}\rangle \big) \\
&= -\int d\mathbf{r}' \int_0^\infty d\omega' \hbar\omega' \langle\{0\}| \mathbf{t}_{\mathrm{DA,D^+A^-}}(\mathbf{r}_{\mathrm{M}},\mathbf{r}',\omega') \cdot \hat{\mathbf{f}}(\mathbf{r}',\omega')\hat{D}_{\mathrm{pol}} |\{0\}\rangle \\
&= -\int d\mathbf{r}' \int_0^\infty d\omega' \hbar\omega' \; \mathbf{t}_{\mathrm{DA,D^+A^-}}(\mathbf{r}_{\mathrm{M}},\mathbf{r}',\omega') \cdot \langle\{0\}| \hat{\mathbf{f}}(\mathbf{r}',\omega')\hat{D}_{\mathrm{pol}} |\{0\}\rangle \\
&= -\int d\mathbf{r}' \int_0^\infty d\omega' \hbar\omega' \; \mathbf{t}_{\mathrm{DA,D^+A^-}}(\mathbf{r}_{\mathrm{M}},\mathbf{r}',\omega') \cdot \langle\{\mathbf{1}(\mathbf{r}',\omega')\}| \hat{D}_{\mathrm{pol}} |\{0\}\rangle \\
&= -\int d\mathbf{r}' \int_0^\infty d\omega' \hbar\omega' \; \mathbf{t}_{\mathrm{DA,D^+A^-}}(\mathbf{r}_{\mathrm{M}},\mathbf{r}',\omega') \cdot \Delta\mathbf{g}^\dagger(\mathbf{r}_{\mathrm{M}},\mathbf{r}',\omega')\exp\big\{-S_{\mathrm{pol}}/2\big\} \\
&= -\int d\mathbf{r}' \int_0^\infty d\omega' \frac{\omega'^3}{c^4\pi\epsilon_0} \mathrm{Im}\epsilon_{\mathrm{r}}(\mathbf{r}',\omega')\boldsymbol{\mu}_{\mathrm{DA,D^+A^-}} \cdot \overline{\overline{\mathbf{G}}}(\mathbf{r}_{\mathrm{M}},\mathbf{r}',\omega') \cdot \overline{\overline{\mathbf{G}}}^*(\mathbf{r}_{\mathrm{M}},\mathbf{r}',\omega') \cdot \Delta\boldsymbol{\mu}_{\mathrm{DA}}\exp\big\{-S_{\mathrm{pol}}/2\big\} \\
&= -\frac{1}{\pi\epsilon_0 c^2} \int_0^\infty d\omega' \; \omega' \Delta\boldsymbol{\mu} \cdot \mathrm{Im}\overline{\overline{\mathbf{G}}}(\mathbf{r}_{\mathrm{M}},\mathbf{r}_{\mathrm{M}},\omega') \cdot \boldsymbol{\mu}_{\mathrm{DA,D^+A^-}}\exp\big\{-S_{\mathrm{pol}}/2\big\} \\
&= -\Lambda_{\mathrm{pol}}\exp\big\{-S_{\mathrm{pol}}/2\big\},
\end{aligned} \tag{S55}$$

where $S_{\mathrm{pol}} \equiv \frac{1}{\hbar c^2\pi\epsilon_0}\int_0^\infty d\omega'\Delta\mu \cdot \mathrm{Im}\overline{\overline{\mathbf{G}}}(\mathbf{r}_{\mathrm{M}},\mathbf{r}_{\mathrm{M}},\omega') \cdot \Delta\mu$ is the polaritonic HR factor.[7] Substituting Eq. S55 into Eq. S54, the term of Λ_{pol} is cancelled out.

$$\begin{aligned}
\hat{\mathcal{V}}^{\{0\},\{0\}}_{\mathrm{DA,D^+A^-}} &= \big((\mathrm{V_{ET}} + \Lambda_{\mathrm{pol}}) \langle\{0\}| \hat{D}_{\mathrm{pol}} |\{0\}\rangle - \langle\{0\}| \boldsymbol{\mu}_{\mathrm{DA,D^+A^-}} \cdot \hat{\mathbf{E}}(\mathbf{r}_{\mathrm{M}})\hat{D}_{\mathrm{pol}} |\{0\}\rangle \big)\hat{D}_{\mathrm{vib}} \\
&= \big((\mathrm{V_{ET}} + \Lambda_{\mathrm{pol}}) \langle\{0\}| \hat{D}_{\mathrm{pol}} |\{0\}\rangle - \Lambda_{\mathrm{pol}}\exp\big\{-S_{\mathrm{pol}}/2\big\}\big)\hat{D}_{\mathrm{vib}} \\
&= \mathrm{V_{ET}}\exp\big\{-S_{\mathrm{pol}}/2\big\}\hat{D}_{\mathrm{vib}}.
\end{aligned} \tag{S56}$$

According to the previous study,[2] k_{Marcus} can be derived as

$$k_{\mathrm{Marcus}} = \frac{|V_{\mathrm{ET}}|^2}{\hbar^2}\exp\big\{-S_{\mathrm{pol}}\big\}\exp\Bigg\{\sum_{u=1}^{m_{\mathrm{vib}}} -(2M_u+1)S_{\mathrm{vib},u}\Bigg\}\int_{-\infty}^{\infty}\mathrm{d}t\exp\big\{\frac{-i\Delta E t}{\hbar}\big\}$$
$$\times\exp\Bigg\{\sum_{u=1}^{m_{\mathrm{vib}}}(M_u+1)S_{\mathrm{vib},u}\exp\big\{-i\omega_{\mathrm{vib},u}t\big\}+M_uS_{\mathrm{vib},u}\exp\big\{i\omega_{\mathrm{vib},u}t\big\}\Bigg\}, \tag{S57}$$

where $M = \langle \hat{b}^\dagger \hat{b}\rangle = (\exp\big\{\hbar\omega_{\mathrm{vib}}/k_{\mathrm{B}}T\big\}-1)^{-1}$. Next, we focus on the Marcus ET rates k_{Marcus} in the limit of low vibrational frequency ($\hbar\omega_{\mathrm{vib},u} \ll k_{\mathrm{B}}T$ for all modes u). In this case, the integrand in Eq. S57 is very short-lived and can be approximated by expanding the exponential up to second order in the $\hbar\omega_{\mathrm{vib},u}$ factors (saddle-point method),[2] which yields

$$k_{\mathrm{Marcus}} \approx \frac{|V_{\mathrm{ET}}|^2}{\hbar}\exp\big\{-S_{\mathrm{pol}}\big\}\sqrt{\frac{\pi}{k_{\mathrm{B}}TE_{\mathrm{r}}}}\exp\big\{-\frac{(\Delta E+E_{\mathrm{r}})^2}{4k_{\mathrm{B}}TE_{\mathrm{r}}}\big\}. \tag{S58}$$

Since S_{pol} is so small that can be neglected,[7] Eq. S58 can be further reduced as

$$k_{\mathrm{Marcus}} \approx \frac{|V_{\mathrm{ET}}|^2}{\hbar}\sqrt{\frac{\pi}{k_{\mathrm{B}}TE_{\mathrm{r}}}}\exp\big\{-\frac{(\Delta E+E_{\mathrm{r}})^2}{4k_{\mathrm{B}}TE_{\mathrm{r}}}\big\}, \tag{S59}$$

which corresponds to the famous rate equation in Marcus ET theory.

S4.2 Derivation of QED-Driven ET Rate

In addition, we can use the similar approach to solve $\kappa_{\mathrm{ET},\{0\}\to\{\mathbf{1}(\mathbf{r},\omega)\}}$. Starting from the Fermi's golden rule,

$$k_{\mathrm{QED}} = \frac{1}{\hbar^2}\int d\mathbf{r}\int_0^\infty d\omega \int_{-\infty}^{\infty} \mathrm{d}t\, \exp\Big\{\frac{-i(\Delta\mathrm{E}+\hbar\omega)t}{\hbar}\Big\}\langle \hat{\mathscr{V}}^{0,\mathbf{1}(\mathbf{r},\omega)}_{\mathrm{DA},\mathrm{D^+A^-}}(t)\hat{\mathscr{V}}^{\mathbf{1}(\mathbf{r},\omega),0}_{\mathrm{D^+A^-},\mathrm{DA}}\rangle. \tag{S60}$$

where

$$\begin{aligned}\hat{\mathscr{V}}^{0,\mathbf{1}(\mathbf{r},\omega)}_{\mathrm{D^+A^-},\mathrm{DA}} &= \langle \mathrm{D^+A^-},\{0\}|\,\hat{\mathscr{V}}_{\mathrm{ET}}\,|\mathrm{DA},\{\mathbf{1}(\mathbf{r},\omega)\}\rangle \\ &= \big((\mathrm{V}_{\mathrm{ET}}+\Lambda_{\mathrm{pol}}\big)\,\langle\{0\}|\,\hat{D}_{\mathrm{pol}}\,|\{\mathbf{1}(\mathbf{r},\omega)\}\rangle - \langle\{0\}|\,\boldsymbol{\mu}_{\mathrm{DA},\mathrm{D^+A^-}}\cdot\hat{\mathbf{E}}(\mathbf{r}_{\mathrm{M}})\hat{D}_{\mathrm{pol}}\,|\{\mathbf{1}(\mathbf{r},\omega)\}\rangle\,\big)\hat{D}_{\mathrm{vib}}.\end{aligned} \tag{S61}$$

Based on Eq. (S61), the formalism of $\kappa_{\mathrm{ET},0\to1_{n'}}$ can be expressed as

$$\begin{aligned}&k_{\mathrm{QED}}\\ &= \frac{1}{\hbar^2}\int d\mathbf{r}\int_0^\infty d\omega|(\mathrm{V}_{\mathrm{ET}}+\Lambda_{\mathrm{pol}}\big)\,\langle\{0\}|\,\hat{D}_{\mathrm{pol}}\,|\{\mathbf{1}(\mathbf{r},\omega)\}\rangle - \langle\{0\}|\,\boldsymbol{\mu}_{\mathrm{DA},\mathrm{D^+A^-}}\cdot\hat{\mathbf{E}}(\mathbf{r}_{\mathrm{M}})\hat{D}_{\mathrm{pol}}\,|\{\mathbf{1}(\mathbf{r},\omega)\}\rangle\,|^2\\ &\times\exp\Big\{\sum_{u=1}^{m_{\mathrm{vib}}} -(2M_u+1)S_{\mathrm{vib},u}\Big\}\int_{-\infty}^{\infty}\mathrm{d}t\exp\Big\{\frac{-i(\Delta\mathrm{E}+\hbar\omega)}{\hbar}\Big\}\\ &\times\exp\Big\{\sum_{u=1}^{m_{\mathrm{vib}}}(M_u+1)S_{\mathrm{vib},u}\exp\big\{-i\omega_{\mathrm{vib},u}t\big\}+M_uS_{\mathrm{vib},u}\exp\big\{i\omega_{\mathrm{vib},u}t\big\}\Big\}\\ &= \frac{2\pi}{\hbar}\int d\mathbf{r}\int_0^\infty d\omega|(\mathrm{V}_{\mathrm{ET}}+\Lambda_{\mathrm{pol}}\big)\,\langle\{0\}|\,\hat{D}_{\mathrm{pol}}\,|\{\mathbf{1}(\mathbf{r},\omega)\}\rangle - \langle\{0\}|\,\boldsymbol{\mu}_{\mathrm{DA},\mathrm{D^+A^-}}\cdot\hat{\mathbf{E}}(\mathbf{r}_{\mathrm{M}})\hat{D}_{\mathrm{pol}}\,|\{\mathbf{1}(\mathbf{r},\omega)\}\rangle\,|^2\\ &\times\rho_{\mathrm{vib}}(\omega),\end{aligned} \tag{S62}$$

where $\rho_{\rm vib}(\omega)$, dubbed vibronic density of states (DOS) or vibrational transition DOS,[6] corresponds to the DOS of molecular degrees of freedoms, and it is defined as

$$\rho_{\rm vib}(\omega) \equiv \frac{1}{2\pi\hbar}\exp\Big\{\sum_{u=1}^{m_{\rm vib}} -(2M_u+1)S_{{\rm vib},u}\Big\}\int_{-\infty}^{\infty}{\rm d}t\ \exp\{\frac{-i(\Delta E+\hbar\omega)t}{\hbar}\}$$
$$\times\exp\Big\{\sum_{u=1}^{m_{\rm vib}}(M_u+1)S_{{\rm vib},u}\exp\{-i\omega_{{\rm vib},u}t\}+M_uS_{{\rm vib},u}\exp\{i\omega_{{\rm vib},u}t\}\Big\}, \qquad \text{(S63)}$$

which is as same as the definition in the QED-ET theory.[6] Next, the coupling term in Eq. S62 can be expanded as

$$\begin{aligned}
&\langle\{0\}|\,\boldsymbol{\mu}_{\rm DA,D^+A^-}\cdot\hat{\mathbf{E}}(\mathbf{r}_{\rm M})\hat{D}_{\rm pol}\,|\{\mathbf{1}(\mathbf{r},\omega)\}\rangle\\
&=\int d\mathbf{r}'\int_0^{\infty}d\omega'\hbar\omega'\mathbf{t}_{\rm DA,D^+A^-}(\mathbf{r}_{\rm M},\mathbf{r}',\omega')\cdot\langle\{\mathbf{1}(\mathbf{r}',\omega')\}|\,\hat{D}_{\rm pol}\,|\{\mathbf{1}(\mathbf{r},\omega)\}\rangle\\
&=\int d\mathbf{r}'\int_0^{\infty}d\omega'\hbar\omega'\mathbf{t}_{\rm DA,D^+A^-}(\mathbf{r}_{\rm M},\mathbf{r}',\omega')\cdot\langle\{\mathbf{1}(\mathbf{r}',\omega')\}|\,\hat{D}_{\rm pol}\,|\{\mathbf{1}(\mathbf{r},\omega)\}\rangle\\
&=\int d\mathbf{r}'\int_0^{\infty}d\omega'\hbar\omega'\mathbf{t}_{\rm DA,D^+A^-}(\mathbf{r}_{\rm M},\mathbf{r}',\omega')\\
&\quad\cdot\big(\delta(\mathbf{r}-\mathbf{r}')\delta(\omega-\omega')-\Delta\mathbf{g}^{\dagger}(\mathbf{r}_{\rm M},\mathbf{r}',\omega')\Delta\mathbf{g}(\mathbf{r}_{\rm M},\mathbf{r},\omega)\big)\exp\{-S_{\rm pol}/2\}\\
&=\big(\hbar\omega\mathbf{t}_{\rm DA,D^+A^-}(\mathbf{r}_{\rm M},\mathbf{r},\omega)-\Lambda_{\rm pol}\Delta\mathbf{g}(\mathbf{r}_{\rm M},\mathbf{r},\omega)\big)\exp\{-S_{\rm pol}/2\}.
\end{aligned} \qquad \text{(S64)}$$

By virtue of Eq. S64, the square term of coupling in Eq. S62 can be expanded as

$$\begin{aligned}
&|({\rm V}_{\rm ET}+\Lambda_{\rm pol})\,\langle\{0\}|\,\hat{D}_{\rm pol}\,|\{\mathbf{1}(\mathbf{r},\omega)\}\rangle-\langle\{0\}|\,\boldsymbol{\mu}_{\rm DA,D^+A^-}\cdot\hat{\mathbf{E}}(\mathbf{r}_{\rm M})\hat{D}_{\rm pol}\,|\{\mathbf{1}(\mathbf{r},\omega)\}\rangle\,|^2\\
&=|-({\rm V}_{\rm ET}+\Lambda_{\rm pol})\Delta\mathbf{g}(\mathbf{r}_{\rm M},\mathbf{r},\omega)+\Lambda_{\rm pol}\Delta\mathbf{g}(\mathbf{r}_{\rm M},\mathbf{r},\omega)-\hbar\omega\mathbf{t}_{\rm DA,D^+A^-}(\mathbf{r}_{\rm M},\mathbf{r},\omega)|^2\exp\{-S_{\rm pol}\}\\
&=|{\rm V}_{\rm ET}\Delta\mathbf{g}(\mathbf{r}_{\rm M},\mathbf{r},\omega)+\hbar\omega\mathbf{t}_{\rm DA,D^+A^-}(\mathbf{r}_{\rm M},\mathbf{r},\omega)|^2\exp\{-S_{\rm pol}\}\\
&=\Big\{|{\rm V}_{\rm ET}\Delta\mathbf{g}(\mathbf{r}_{\rm M},\mathbf{r},\omega)|^2+(\hbar\omega)^2|\mathbf{t}_{\rm DA,D^+A^-}(\mathbf{r}_{\rm M},\mathbf{r},\omega)|^2\\
&\qquad+\hbar\omega{\rm V}_{\rm ET}(\mathbf{t}_{\rm DA,D^+A^-}(\mathbf{r}_{\rm M},\mathbf{r},\omega)\cdot\Delta\mathbf{g}^{\dagger}(\mathbf{r}_{\rm M},\mathbf{r},\omega)+{\rm h.c.})\Big\}\exp\{-S_{\rm pol}\}.
\end{aligned} \qquad \text{(S65)}$$

According to Eq. S65, k_{QED} is derived as follows.

$$
\begin{aligned}
&k_{\mathrm{QED}} \\
&= \frac{2\pi}{\hbar}\int_0^{\infty} d\omega\ \rho_{\mathrm{vib}}(\omega)\ \exp\{-S_{\mathrm{pol}}\}\frac{1}{\hbar c^2\pi\epsilon_0}\Big\{{\mathrm{V}_{\mathrm{ET}}}^2\boldsymbol{\Delta\mu}\cdot\mathrm{Im}\overline{\overline{\mathbf{G}}}(\mathbf{r}_{\mathrm{M}},\mathbf{r}_{\mathrm{M}},\omega)\cdot\boldsymbol{\Delta\mu} \\
&\quad -2\mathrm{V}_{\mathrm{ET}}\hbar\omega\boldsymbol{\mu}_{\mathrm{DA,D^+A^-}}\cdot\mathrm{Im}\overline{\overline{\mathbf{G}}}(\mathbf{r}_{\mathrm{M}},\mathbf{r}_{\mathrm{M}},\omega)\cdot\boldsymbol{\Delta\mu}+\hbar^2\omega^2\boldsymbol{\mu}_{\mathrm{DA,D^+A^-}}\cdot\mathrm{Im}\overline{\overline{\mathbf{G}}}(\mathbf{r}_{\mathrm{M}},\mathbf{r}_{\mathrm{M}},\omega)\cdot\boldsymbol{\mu}_{\mathrm{DA,D^+A^-}}\Big\} \\
&= \frac{2\pi}{\hbar}\int_0^{\infty} d\omega\ J_{\mathrm{pol}}(\omega)\rho_{\mathrm{vib}}(\omega),
\end{aligned}
\tag{S66}
$$

which correspond to Eq. 2 in the manuscript. $J_{\mathrm{pol}}(\omega)$ corresponds to the polaritonic spectral density, and it is defined as

$$
\begin{aligned}
&J_{\mathrm{pol}}(\omega) \\
&\equiv \exp\{-S_{\mathrm{pol}}\}\frac{1}{\hbar c^2\pi\epsilon_0}\Big\{{\mathrm{V}_{\mathrm{ET}}}^2\Delta\boldsymbol{\mu}\cdot\mathrm{Im}\overline{\overline{\mathbf{G}}}(\mathbf{r}_{\mathrm{M}},\mathbf{r}_{\mathrm{M}},\omega)\cdot\boldsymbol{\Delta\mu} \\
&\quad +2\mathrm{V}_{\mathrm{ET}}\hbar\omega\boldsymbol{\mu}_{\mathrm{DA,D^+A^-}}\cdot\mathrm{Im}\overline{\overline{\mathbf{G}}}(\mathbf{r}_{\mathrm{M}},\mathbf{r}_{\mathrm{M}},\omega)\cdot\boldsymbol{\Delta\mu}+\hbar^2\omega^2\boldsymbol{\mu}_{\mathrm{DA,D^+A^-}}\cdot\mathrm{Im}\overline{\overline{\mathbf{G}}}(\mathbf{r}_{\mathrm{M}},\mathbf{r}_{\mathrm{M}},\omega)\cdot\boldsymbol{\mu}_{\mathrm{DA,D^+A^-}}\Big\} \\
&= \exp\{-S_{\mathrm{pol}}\}\Big\{{\mathrm{V}_{\mathrm{ET}}}^2\tilde{S}_{\mathrm{pol}}(\omega) \\
&\quad +2\mathrm{V}_{\mathrm{ET}}\tilde{\Lambda}_{\mathrm{pol}}(\omega)+\frac{\hbar}{c^2\pi\epsilon_0}\omega^2\boldsymbol{\mu}_{\mathrm{DA,D^+A^-}}\cdot\mathrm{Im}\overline{\overline{\mathbf{G}}}(\mathbf{r}_{\mathrm{M}},\mathbf{r}_{\mathrm{M}},\omega)\cdot\boldsymbol{\mu}_{\mathrm{DA,D^+A^-}}\Big\},
\end{aligned}
\tag{S67}
$$

where $\tilde{S}_{\mathrm{pol}}(\omega)$ and $\tilde{\Lambda}_{\mathrm{pol}}(\omega)$ correspond to the spectral distribution of the polaritonic Huang-Rhys factor and reorganization dipole self-coupling, respectively. Since our previous study showed that S_{pol} and Λ_{pol} are extremely small even under strong light-matter coupling regime,[7] the contribution from S_{pol}, $\tilde{S}_{\mathrm{pol}}(\omega)$ and $\tilde{\Lambda}_{\mathrm{pol}}(\omega)$ are omitted, leading to

$$
J_{\mathrm{pol}}(\omega)\approx\frac{\hbar}{c^2\pi\epsilon_0}\omega^2\boldsymbol{\mu}_{\mathrm{DA,D^+A^-}}\cdot\mathrm{Im}\overline{\overline{\mathbf{G}}}(\mathbf{r}_{\mathrm{M}},\mathbf{r}_{\mathrm{M}},\omega)\cdot\boldsymbol{\mu}_{\mathrm{DA,D^+A^-}}. \tag{S68}
$$

Notably, Eq. S68 is associated with the light-matter coupling strength g_0 in our previous study.[7] Eq. S68 is the working equation of calculating k_{QED} in this work.

S5 Conversion of mQED-ET Theory to Cavity QED-ET Theory

According to our previous study, the imaginary part of single-point dyadic Green's function under the condition of a single photonic mode can be expressed as[7,10]

$$\mathrm{Im}\overline{\overline{\mathbf{G}}}(\mathbf{r}_\alpha, \mathbf{r}_\alpha, \omega) = \frac{\pi c^2}{2\omega V_{\mathrm{eff,s}}} \boldsymbol{\epsilon}_{\mathrm{s}}(\mathbf{r}_\alpha, \omega_{\mathrm{s}}) \otimes \boldsymbol{\epsilon}_{\mathrm{s}}(\mathbf{r}_\alpha, \omega_{\mathrm{s}}) \delta(\omega - \omega_{\mathrm{s}}), \tag{S69}$$

where $V_{\mathrm{eff,s}}$ and ω_{s} stand for the effective mode volume and frequency of a single photonic mode. Next, substituting Eq. S69 into the $\mathrm{Im}\overline{\overline{\mathbf{G}}}(\mathbf{r}_{\mathrm{M}}, \mathbf{r}_{\mathrm{M}}, \omega)$ in Eq. S68, we obtain

$$\begin{aligned} J_{\mathrm{pol}}(\omega) &= \frac{\hbar}{c^2 \pi \epsilon_0} \omega^2 \frac{\pi c^2}{2\omega V_{\mathrm{eff,s}}} |\boldsymbol{\mu}_{\mathrm{DA,D^+A^-}} \cdot \boldsymbol{\epsilon}_{\mathrm{s}}(\mathbf{r}_\alpha, \omega_{\mathrm{s}})|^2 \delta(\omega - \omega_{\mathrm{s}}) \\ &= \frac{\hbar\omega}{2\epsilon_0 V_{\mathrm{eff,s}}} |\boldsymbol{\mu}_{\mathrm{DA,D^+A^-}} \cdot \boldsymbol{\epsilon}_{\mathrm{s}}(\mathbf{r}_\alpha, \omega_{\mathrm{s}})|^2 \delta(\omega - \omega_{\mathrm{s}}). \end{aligned} \tag{S70}$$

Substituting Eq. S70 into the $J_{\mathrm{pol}}(\omega)$ in Eq. S66, we obtain the QED-driven ET rates considering the single photonic mode.

$$k_{\mathrm{QED}} = \frac{2\pi}{\hbar} \frac{\hbar\omega_{\mathrm{s}}}{2\epsilon_0 V_{\mathrm{eff,s}}} |\boldsymbol{\mu}_{\mathrm{DA,D^+A^-}} \cdot \boldsymbol{\epsilon}_{\mathrm{s}}(\mathbf{r}_\alpha, \omega_{\mathrm{s}})|^2 \times \rho_{\mathrm{vib}}(\omega_{\mathrm{s}}). \tag{S71}$$

Combining Eq. S71 and Eq. S59 and apply the limit of low vibrational frequency, the total ET rates in the framework of cavity QED is derived.

$$\begin{aligned} k_{\mathrm{ET}} =& \sqrt{\frac{\pi}{\hbar^2 k_{\mathrm{B}} T E_{\mathrm{r}}}} \big(|V_{\mathrm{ET}}|^2 \exp\{-\frac{(\Delta E + E_{\mathrm{r}})^2}{4 k_{\mathrm{B}} T E_{\mathrm{r}}}\} \\ &+ \frac{\hbar\omega_{\mathrm{s}}}{2\epsilon_0 V_{\mathrm{eff,s}}} |\boldsymbol{\mu}_{\mathrm{DA,D^+A^-}} \cdot \boldsymbol{\epsilon}_{\mathrm{s}}(\mathbf{r}_\alpha, \omega_{\mathrm{s}})|^2 \exp\{-\frac{(\Delta E + \hbar\omega_{\mathrm{s}} + E_{\mathrm{r}})^2}{4 k_{\mathrm{B}} T E_{\mathrm{r}}}\} \big), \end{aligned} \tag{S72}$$

which corresponds to the working equation (Eq. 38) in the article of the Semenov-Nitzan theory considering the case of slow electron and fast cavity mode.[1] Note that the the conditions for the slow electron and fast cavity mode indicate that the cavity mode does not

affect the vibrational degrees of freedom, which is consistent with the Condon approximation (Eq. S7).

S6 Conversion of mQED-ET Theory to Vacuum QED-ET Theory

In free space, $\mathrm{Im}\overline{\overline{\mathbf{G}}}(\mathbf{r}_\mathrm{M}, \mathbf{r}_\mathrm{M}, \omega)$ is written as[5,10]

$$\mathrm{Im}\overline{\overline{\mathbf{G}}}(\mathbf{r}_\mathrm{M}, \mathbf{r}_\mathrm{M}, \omega) = \frac{\omega}{6\pi c}\overline{\overline{\mathbf{I}}}_3. \tag{S73}$$

Substituting Eq. S73 into the $\mathrm{Im}\overline{\overline{\mathbf{G}}}(\mathbf{r}_\mathrm{M}, \mathbf{r}_\mathrm{M}, \omega)$ in Eq. S68, we obtain

$$\begin{aligned} J_\mathrm{pol}(\omega) &= \frac{\hbar}{6c^3\pi^2\epsilon_0}\omega^3|\boldsymbol{\mu}_{\mathrm{DA,D^+A^-}}|^2 \\ &= \frac{\hbar\omega|\boldsymbol{\mu}_{\mathrm{DA,D^+A^-}}|^2}{6\epsilon_0}\frac{\omega^2}{\pi^2c^3} \\ &= F(\omega) \times \rho_\mathrm{ph}(\omega), \end{aligned} \tag{S74}$$

where $F(\omega) = \hbar\omega|\boldsymbol{\mu}_{\mathrm{DA,D^+A^-}}|^2/(6\varepsilon_0)$ corresponds to the light-matter coupling factor and $\rho_\mathrm{ph}(\omega) = \omega^2/(\pi^2c^3)$ represents the the photonic DOS in free space. Substituting Eq. S74 into the J_pol in Eq. S66, the QED-ET theory is derived.[6]

$$k_\mathrm{QED} = \frac{2\pi}{\hbar}\int_0^\infty \mathrm{d}\omega F(\omega) \times \rho_\mathrm{ph}(\omega) \times \rho_\mathrm{vib}(\omega). \tag{S75}$$

S7 Supporting Figures

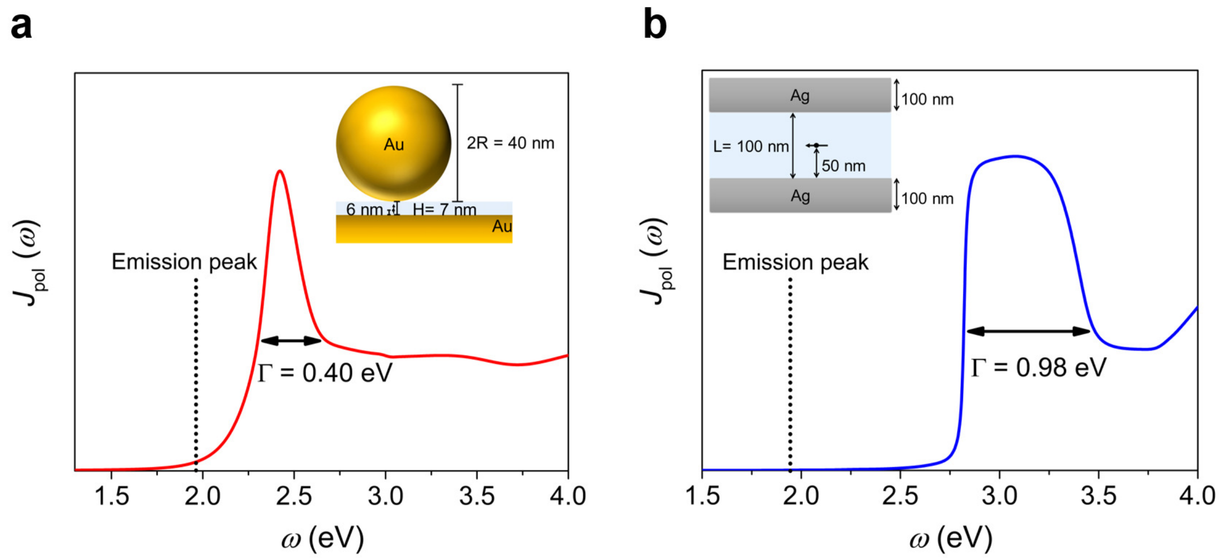

Figure S1: Polaritonic spectral density $J_{\mathrm{pol}}(\omega)$ of (a) the plasmonic cavity and (b) the photonic cavity. The photonic dissipation is estimated by the peak widths. According to our previous study, the integral of J_{pol} corresponds to the light-matter coupling strength g_0. Based on this method, $g_0 = 2.15 \times 10^{-2}$ eV in (a) and $g_0 = 2.87 \times 10^{-4}$ eV in (b), which are far smaller than the corresponding peak widths.

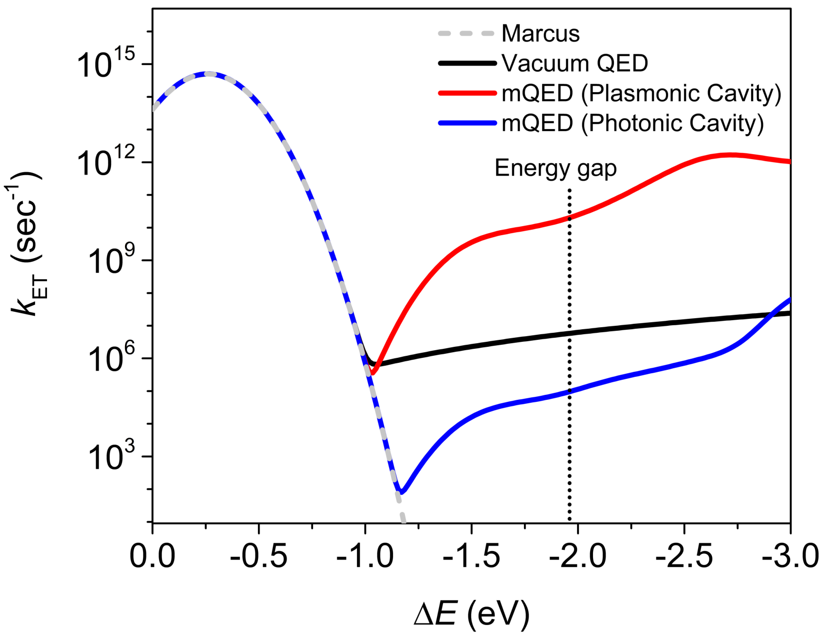

Figure S2: Energy gap dependence of ET rates. The dotted line indicates the energy gap of BODIPY-TPA.[11] The following parameters were used: $|\boldsymbol{\mu}_{\mathrm{DA,D^+A^-}}| = 2.67$ Debye, $T = 300$ K, $E_\mathrm{r} = 0.26$ eV and $\mathrm{V_{ET}} = 1000\ \mathrm{cm}^{-1}$.[11] The cavities structures are those used in Fig. S1.

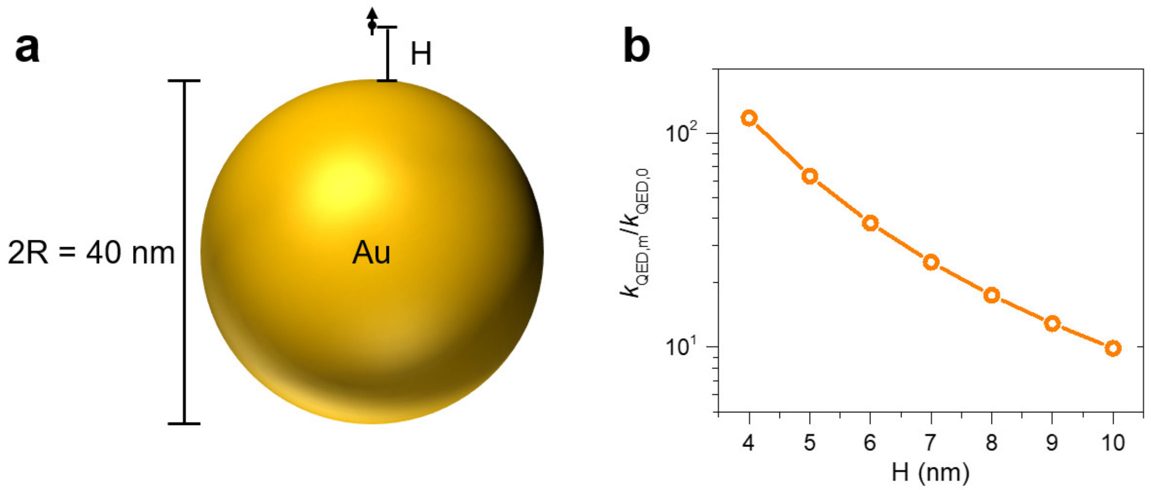

Figure S3: ET rate enhancements in a gold nanosphere system. The black arrow indicates the molecular transition dipole's position and direction. The position is directly on top of the gold nanosphere. H represents the molecular height above the particle surface. The direction of the transition dipole is along the radial direction. (b) Height dependence of the ET rate enhancements. The parameters of $\rho_{\text{vib}}(\omega)$ follow the ones in Fig. 3.

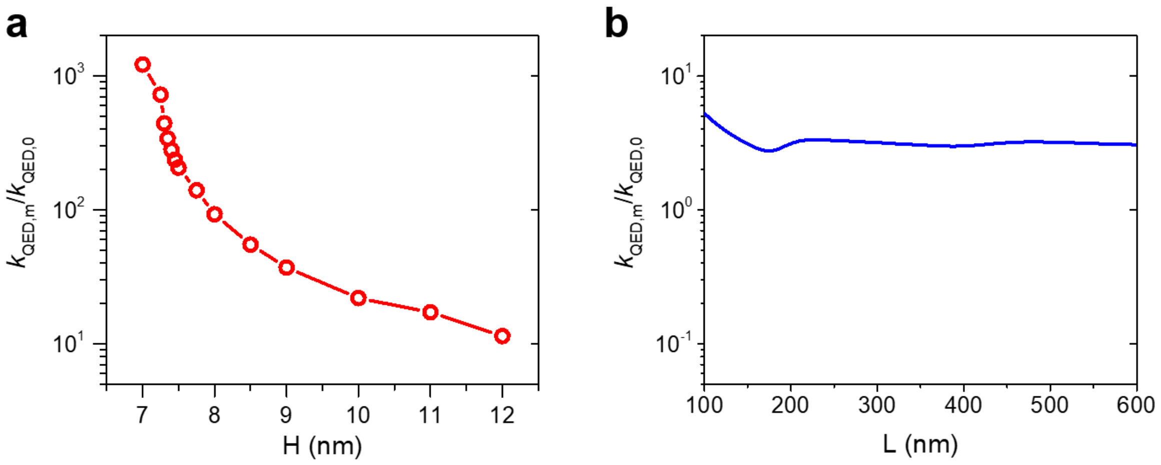

Figure S4: QED-driven ET rate enhancements for a randomly oriented molecule in (a) the plasmonic cavity and (b) the photonic cavity. The cavity structures are the same as those in Fig. 3.

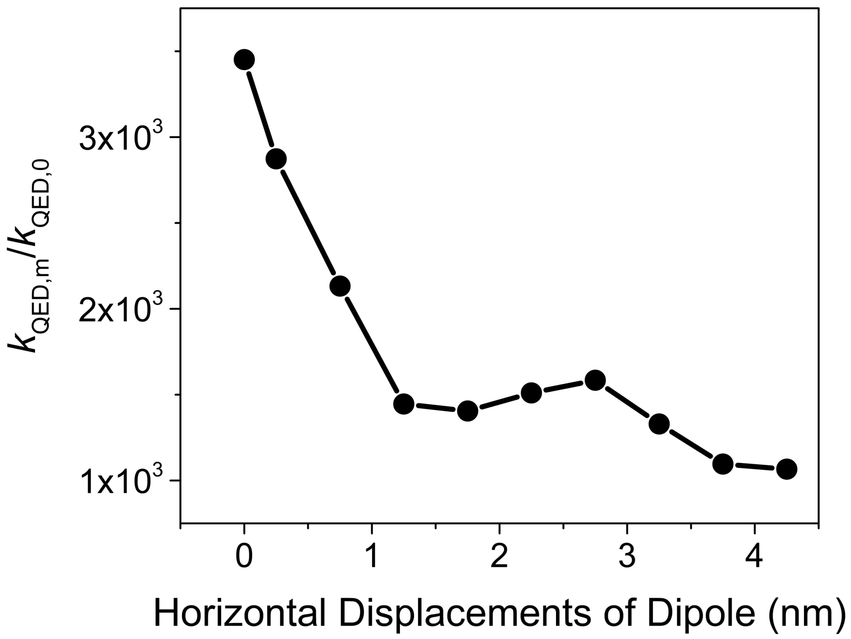

Figure S5: QED-driven ET rate enhancements for a single vertical dipole positioned 6 nm above the surface in the plasmonic cavity at various horizontal radial distances. The origin is defined as the position directly beneath the sphere. The cavity structures are the same as those in Fig. 3.

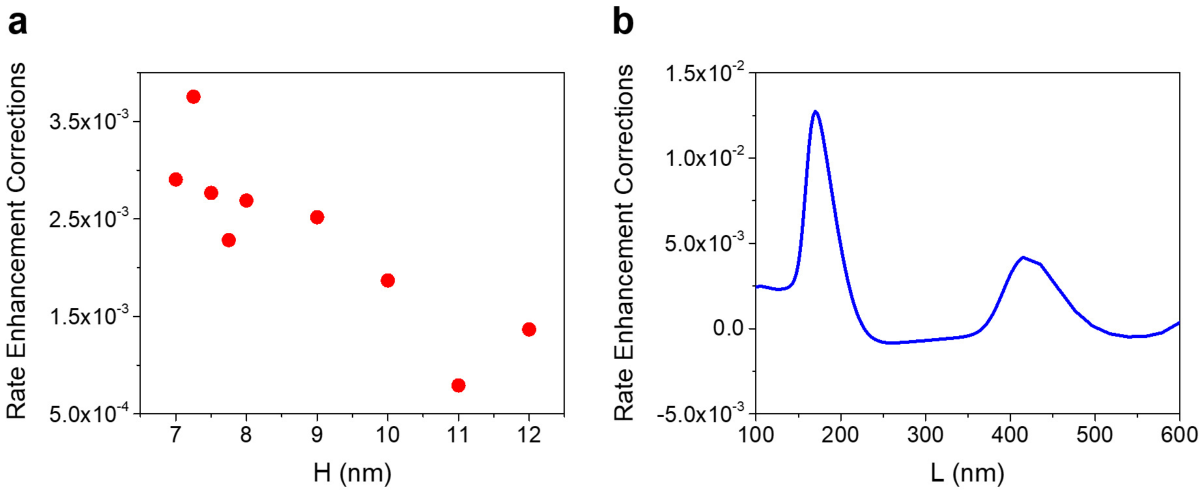

Figure S6: Rate enhancement corrections in (a) the plasmonic cavity and (b) the photonic cavity. The cavity structures are the ones in Fig. 3. The definition of the rate enhancement corrections is $(k_{\text{QED,m,approx}}/k_{\text{QED,0,approx}} - k_{\text{QED,m,exact}}/k_{\text{QED,0,exact}})/(k_{\text{QED,m,exact}}/k_{\text{QED,0,exact}})$, where $k_{\text{QED,m,approx}}$ ($k_{\text{QED,m,approx}}$) is the approximated QED-driven ET rate in a medium (vacuum) without considering permanent dipole difference and dipole self-energy and $k_{\text{QED,m,exact}}$ ($k_{\text{QED,m,exact}}$) is the exact solution of QED-driven ET rate in a medium (vacuum).

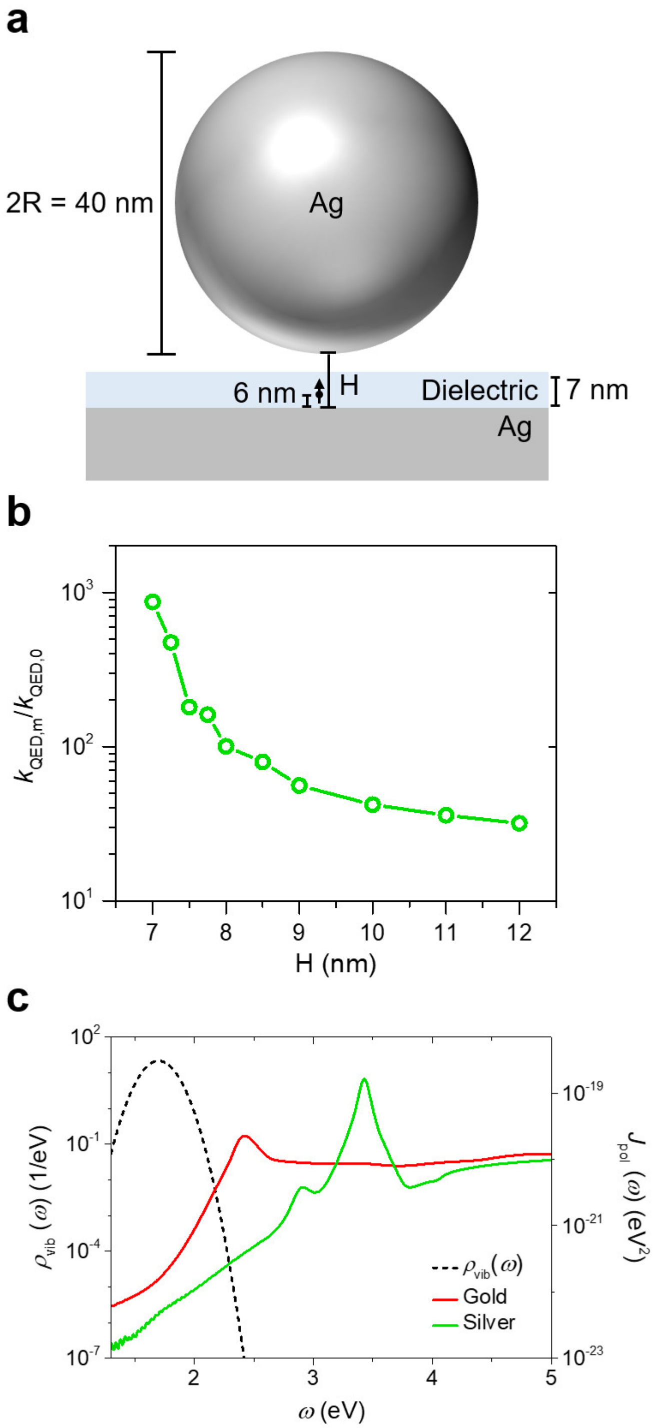

Figure S7: Effects of different materials on QED-driven ET rate enhancements. (a) Schematic illustration of the silver plasmonic cavity, whose size is as same as the gold one. (b) QED-driven ET rate enhancements in the silver plasmonic cavity. (c) Electron-transfer overlap in the gold and silver plasmonic cavities. The parameter H = 7 nm is applied for the two cavities. The parameters of $\rho_{\mathrm{vib}}(\omega)$ follow the ones in Fig. 3.

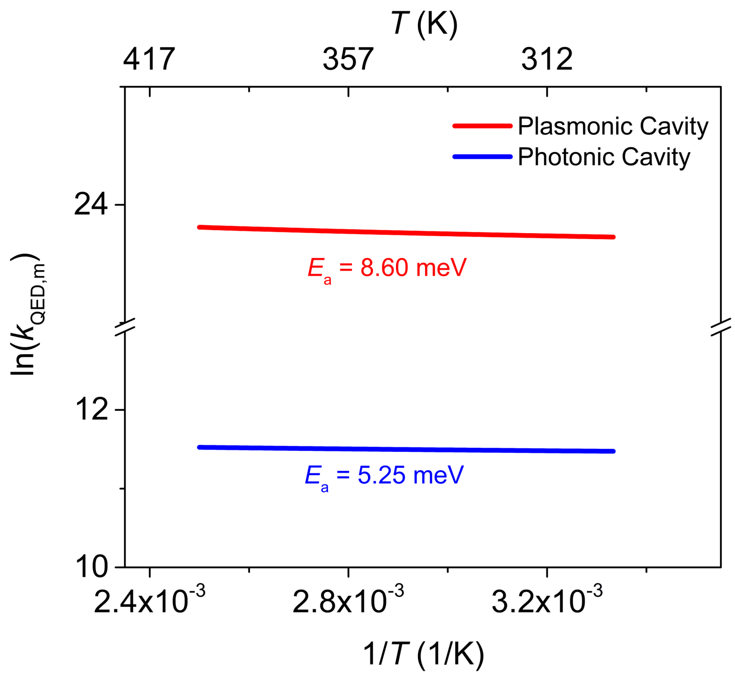

Figure S8: Arrhenius plot of BODIPY-TPA in the plasmonic cavity (red line) and the photonic cavity (blue line). The cavities structures are those used in Fig. S1. The following parameters are applied: ET transition dipole $|\mu_{\mathrm{DA,D^+A^-}}| = 2.67$ Debye, $\Delta E = 1.96$ eV and $E_{\mathrm{r}} = 0.26$ eV.

References

(1) Semenov, A.; Nitzan, A. Electron Transfer in Confined Electromagnetic Fields. *J. Chem. Phys.* **2019**, *150*, 174122.

(2) Nitzan, A. *Chemical Dynamics in Condensed Phases: Relaxation, Transfer and Reactions in Condensed Molecular Systems*; Oxford university press, 2006.

(3) Gruner, T.; Welsch, D.-G. Green-Function Approach to the Radiation-Field Quantization for Homogeneous and Inhomogeneous Kramers-Kronig Dielectrics. *Phys. Rev. A* **1996**, *53*, 1818.

(4) Dung, H. T.; Knöll, L.; Welsch, D.-G. Three-Dimensional Quantization of the Electromagnetic Field in Dispersive and Absorbing Inhomogeneous Dielectrics. *Phys. Rev. A* **1998**, *57*, 3931.

(5) Buhmann, S. Y. *Dispersion Forces I: Macroscopic quantum electrodynamics and ground-state Casimir, Casimir–Polder and Van Der Waals forces*; Springer, 2013.

(6) Wei, Y.-C.; Hsu, L.-Y. Cavity-Free Quantum-Electrodynamic Electron Transfer Reactions. *J. Phys. Chem. Lett.* **2022**, *13*, 9695–9702.

(7) Wei, Y.-C.; Hsu, L.-Y. Polaritonic Huang–Rhys Factor: Basic Concepts and Quantifying Light–Matter Interactions in Media. *J. Phys. Chem. Lett.* **2023**, *14*, 2395–2401.

(8) Chang, H.-T.; Zhang, P.-P.; Cheng, Y.-C. Criteria for the Accuracy of Small Polaron Quantum Master Equation in Simulating Excitation Energy Transfer Dynamics. *J. Chem. Phys.* **2013**, *139*, 224112.

(9) Hsu, L.-Y.; Tsai, T.-W.; Jin, B.-Y. Transport through a Mixed-Valence Molecular Transistor in the Sequential-Tunneling Regime: Theoretical Insight from the Two-Site Peierls–Hubbard Model. *J. Chem. Phys.* **2010**, *133*, 144705.

(10) Novotny, L.; Hecht, B. *Principles of Nano-Optics*; Cambridge university press, 2012.

(11) Buck, J. T.; Wilson, R. W.; Mani, T. Intramolecular Long-Range Charge-Transfer Emission in Donor–Bridge–Acceptor Systems. *J. Phys. Chem. Lett.* **2019**, *10*, 3080–3086.
